# *Neisseria meningitidis* Type IV Pili Composed of Sequence Invariable Pilins Are Masked by Multisite Glycosylation

**DOI:** 10.1371/journal.ppat.1005162

**Published:** 2015-09-14

**Authors:** Joseph Gault, Mathias Ferber, Silke Machata, Anne-Flore Imhaus, Christian Malosse, Arthur Charles-Orszag, Corinne Millien, Guillaume Bouvier, Benjamin Bardiaux, Gérard Péhau-Arnaudet, Kelly Klinge, Isabelle Podglajen, Marie Cécile Ploy, H. Steven Seifert, Michael Nilges, Julia Chamot-Rooke, Guillaume Duménil

**Affiliations:** 1 Structural Mass Spectrometry and Proteomics Unit, Institut Pasteur, CNRS UMR 3528, Paris, France; 2 Institut Pasteur, Unité de Bioinformatique Structurale, CNRS UMR 3528, Département de Biologie Structurale et Chimie, Paris, France; 3 INSERM, U970, Paris Cardiovascular Research Center, Paris, France; 4 Université Paris Descartes, Faculté de Médecine Paris Descartes, Paris, France; 5 CNRS, UMR3528, Paris, France; 6 Department of Microbiology-Immunology, Northwestern University Feinberg School of Medicine, Chicago, Illinois, United States of America; 7 Service de Microbiologie, Assistance Publique-Hôpitaux de Paris, Hôpital Européen Georges-Pompidou, Paris, France; 8 INSERM UMR1092, Faculté de Médecine, Université de Limoges, Limoges, France; Osaka University, JAPAN

## Abstract

The ability of pathogens to cause disease depends on their aptitude to escape the immune system. Type IV pili are extracellular filamentous virulence factors composed of pilin monomers and frequently expressed by bacterial pathogens. As such they are major targets for the host immune system. In the human pathogen *Neisseria meningitidis*, strains expressing class I pilins contain a genetic recombination system that promotes variation of the pilin sequence and is thought to aid immune escape. However, numerous hypervirulent clinical isolates express class II pilins that lack this property. This raises the question of how they evade immunity targeting type IV pili. As glycosylation is a possible source of antigenic variation it was investigated using top-down mass spectrometry to provide the highest molecular precision on the modified proteins. Unlike class I pilins that carry a single glycan, we found that class II pilins display up to 5 glycosylation sites per monomer on the pilus surface. Swapping of pilin class and genetic background shows that the pilin primary structure determines multisite glycosylation while the genetic background determines the nature of the glycans. Absence of glycosylation in class II pilins affects pilus biogenesis or enhances pilus-dependent aggregation in a strain specific fashion highlighting the extensive functional impact of multisite glycosylation. Finally, molecular modeling shows that glycans cover the surface of class II pilins and strongly decrease antibody access to the polypeptide chain. This strongly supports a model where strains expressing class II pilins evade the immune system by changing their sugar structure rather than pilin primary structure. Overall these results show that sequence invariable class II pilins are cloaked in glycans with extensive functional and immunological consequences.

## Author Summary

During infection pathogens and their host engage in a series of measures and counter-measures to promote their own survival: pathogens express virulence factors, the immune system targets these surface structures and pathogens modify them to evade detection. Like numerous bacterial pathogens, *Neisseria meningitidis* express type IV pili, long filamentous adhesive structures composed of pilins. Intriguingly the amino acid sequences of pilins from most hypervirulent strains do not vary, raising the question of how they evade the immune system. This study shows that the pilus structure is completely coated with sugars thus limiting access of antibodies to the pilin polypeptide chain. We propose that multisite glycosylation and thus variation in the type of sugar mediates immune evasion in these strains.

## Introduction

Members of the *Neisseria* genus are Gram-negative proteobacteria that include several commensals such as *N*. *sicca*, *N*. *lactamica* or *N*. *elongata* and two human pathogens, *N*. *gonorrheae* and *N*. *meningitidis*. Both of these are highly adapted for interaction with humans, their unique host. *N*. *gonorrheae* colonizes the human urogenital tract and is responsible for a sexually transmitted infection characterized by a massive inflammatory response and purulent discharge. *Neisseria meningitis* is responsible for devastating sepsis and meningitis [1]. *N*. *meningitidis* proliferates on the surface of epithelial cells lining the nasopharynx in approximately 5 to 30% of the total human population. Pathogenesis is initiated when bacteria access the bloodstream from the throat, survive and multiply in the blood. Systemic infection and perturbation of vascular function lead to sepsis, the most severe form of the disease associated with organ dysfunction, limb necrosis and death in certain cases. *N*. *meningitidis* can also cross the blood-brain barrier and access the cerebrospinal fluid, leading to meningitis.

Type IV pili (Tfp) are extracellular filamentous organelles that can be found on a large number of bacterial species [2]. In the case of *Neisseria spp*. they are key virulence factors. These abundant structures are 6–8 nm wide, can measure several microns in length and are expressed by all pathogenic *Neisseria spp*. strains. Type IV pili are primarily composed of a single protein or major pilin, called PilE in *Neisseria spp*., which is assembled in a polymeric helical fiber. *Neisseria* type IV pilins have been grouped in two classes (class I and class II) based on the recognition of the SM1 antibody. This antibody reacts with the linear epitope E^49^YYLN^53^, which is specific to class I pilins [3]. It was later recognized that the genomic location of the class I and II pilin genes are also different [4, 5]. Type IV pili provide several properties to the bacteria: auto-aggregation, adhesion to host cells, intracellular signaling, competence and a form of motility called twitching motility [6]. The importance of this structure during *N*. *gonorrheae* infection has been demonstrated in human volunteers [7]. Male volunteers inoculated with a type IV pili deficient strain only developed a watery urethral discharge or none at all. More recently, using mice grafted with human skin, Melican *et al*. showed that type IV pili mediated adhesion of *N*. *meningitidis* is a determining factor in vascular damage observed during *purpura fulminans* [8].

As a countermeasure against this virulence factor the immune system produces antibodies against type IV pili [9]. The efficacy of *N*. *meningitidis* to proliferate in the throat and in blood during productive infection thus depends on its ability to evade type IV pili specific antibodies. The amino acid sequence of class I pilins can vary by a process called antigenic variation [10]. Beside the expression locus of the major pilins a variable number of non-expressed (silent) *pilS* loci with different but homologous sequences are present in *Neisseria spp*. genomes. Pilin antigenic variation results from a gene conversion, which transfers DNA from the silent cassettes to the expression locus. Thus, the pilin sequence can change generating multiple different antigens. Surprisingly, it was recently recognized that pilins belonging to class II lack this antigenic variation in *Neisseria meningitidis* [11, 12]. Strains with sequence invariable *pilE* genes are frequently isolated worldwide independently of serogroup, year or country of isolation [5]. Interestingly class II pilin genes are restricted to certain clonal complexes, and all pilin genes from clonal complexes cc1, cc5, cc8, cc11 and cc174 are class II. Importantly, these clonal complexes display among the highest disease to carriage ratio, in other words they are hypervirulent [13]. Another interesting feature of these clonal complexes is the association with epidemic meningococcal disease (cc1, cc5 and cc11). Countries in the “meningitis belt” in sub-Saharan Africa have the highest burden of meningococcal disease with both large seasonal epidemics, and much higher incidence rates compared to other areas of the world where outbreaks are small and sporadic. These studies therefore raise the question of how, in absence of primary structure variation, do class II expressing strains evade immunity targeted against type IV pili?

Another potential source of surface variation is post translational modification and in particular glycosylation. Pilin glycosylation has been identified in strains expressing class I pilins that display a single glycosylation site on Ser^63^ [14–17] but has never been studied in class II pilin expressing strains. Importantly, genes involved in the glycosylation of surface structures (*pgl* genes) are submitted to phase variation. As a consequence, oligosaccharides present on the bacterial surface vary between strains and change for one strain during the course of nasopharynx colonization and infection. The *Neisseria spp*. glycosylation pathway starts with the synthesis of an undecaprenyl diphosphate (Undpp) monosaccharide in the cytoplasm. Three enzymes are involved in this step, PglB, C and D. In strains expressing the *pglB1* allele these enzymes synthesize an undecaprenyl-DATDH (diacetamido trideoxyhexose) and in strains expressing the *pglB2* allele a GATDH (glyceramido trideoxyhexose) core [18]. The Unpp-monosaccharide can then be modified with additional sugars by three glycosyltransferases *pglH*, *pglA and pglE*, the latter two being submitted to phase variation. When in the ON-phase, *pglA* leads to the addition of a galactosyl residue on GATDH or DATDH [19]. PglH adds a glycosyl residue on the same site [20]. Recently a *pglH2* allele was described whose product adds an N-acetyl glucosamine residue on the first sugar [21]. When a disaccharide is formed a third sugar can be added by the PglE transferase. PglF is then responsible for the translocation of this structure to the periplasmic side of the inner membrane [22]. Finally, the PglO/L oligosaccharide transferase adds the sugar chain onto the pilin [23, 24].

Given the clinical importance of strains expressing class II pilins and the invariable nature of their sequences, we decided to explore how such strains could evade immunity directed against type IV pili. Since pilin glycosylation is a potential source of surface structure variation we determined the nature of class II pilin glycosylation and show that this could provide immune escape in the absence of primary structure variation.

## Results

### The class II pilin from the FAM20 strain displays multiple glycosylation sites

The prototypical strain expressing a class II pilin is the FAM18 strain that was isolated from the cerebrospinal fluid of a patient in North Carolina USA in the 1980s. Its genome has been sequenced and is publicly available [25]. We used a Nalidixic acid resistant variant of this strain called FAM20 to characterize the posttranslational modifications of this representative class II pilin [26]. Type IV pili were purified and characterized using a combination of high-resolution mass profiling and top-down mass spectrometry [27].

**Fig 1:**
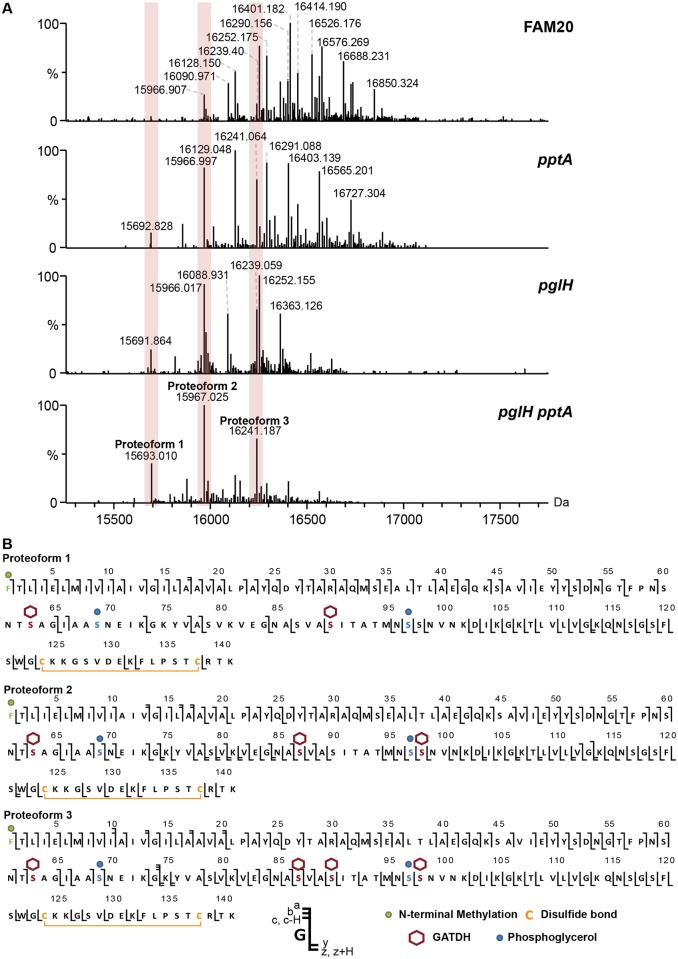
Analysis of the posttranslational modifications of the class II pilin expressing strain FAM20. (A) Whole protein analysis of type IV pili prepared from the FAM20 strain and mutants of the same strain *pptA*, *pglH* and the double mutant *pglHpptA*. (B) Top-down fragmentation maps of the three main proteoforms found in the FAM20*pglHpptA* strain showing identified PTMs localized to sites on the PilE primary structure.

Mass profiling of the FAM20 strain produced an exceptionally complex spectrum with over 20 different proteoforms [28] clearly distinguishable (Fig 1A, FAM20). Pilin masses ranged from 15967 Da to over 16850 Da while the molecular mass predicted from the genome is only 14524 Da strongly indicating that numerous post translational modifications (PTMs) were present. Pilin sequences from individual clones were identical indicating that differences in mass were not due to recombination at the pilin locus as expected from previous studies [12]. In order to identify the different PTMs on this pilin we proceeded to simplify the spectral pattern using specific mutants deficient for genes involved in PTMs. Certain proteoforms of the FAM20 major pilin were separated by 124 Da suggesting that phosphoethanolamine (PE) was present. As *pptA* is responsible for PE modification a *pptA* deletion mutant was generated (Fig 1A, *pptA*) and type IV pili were purified from this strain [29, 30]. The overall pattern of pilin purified from the FAM20*pptA* strain was shifted towards lower masses and the number of major proteoforms was reduced to about 12. Peaks with differences of mass corresponding to one hexose were also frequently observed in the spectra (162 Da). Since FAM18 PglH was found to add a glucose residue onto DATDH (glycosyl transferase) we generated and tested a *pglH* mutant [20]. The complexity of the spectrum obtained with the *pglH* strain was also greatly reduced confirming the activity of this enzyme (Fig 1A, *pglH*). To combine the effects of each mutation a double mutant was made. The *pglHpptA* double mutant generated pili with only 3 major proteoforms (15693, 15967 and 16241 Da). Strikingly, the mass difference between the 3 peaks corresponded to one GATDH moiety (274 Da, Fig 1, *pglHpptA*). This result is a strong indication that the FAM20 pilin is glycosylated at least at 3 sites in contrast with previously analyzed strains that showed only one glycosylation site [18, 23].

**Fig 2:**
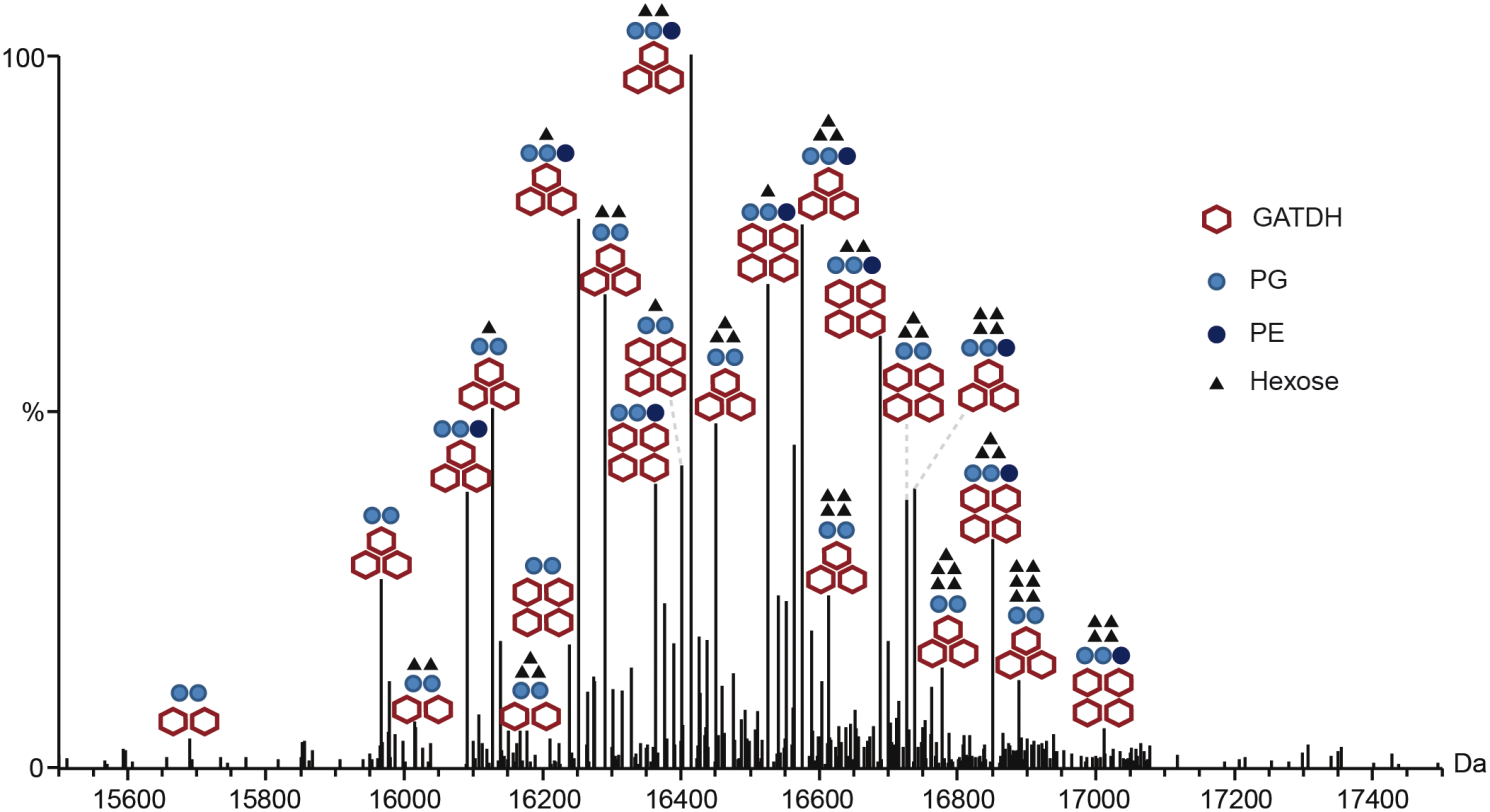
Complete description of the 23 proteoforms expressed by the FAM20 strain.

The reduced complexity of the *pptApglH* double mutant allowed us to identify the PTMs found on the 3 proteoforms using top-down mass spectrometry (Fig 1B, S1 Fig and S1 Table). Different charge states corresponding to each proteoform were submitted to Electron Transfer Dissociation (ETD) fragmentation. As expected, the 3 proteoforms were found to be modified with the usual pilin modifications: N-terminal methylation, disulfide bond between the two cysteine residues and two phosphoglycerol moieties on Ser^69^ and Ser^97^ (Table 1). Proteoform 1 displayed two glycosylation sites with GATDH at Ser^63^ and Ser^90^. Proteoform 2 harbored three glycosylation sites at Ser^63^, Ser^87^ and Ser^98^. Proteoform 3 harbored a fourth glycosylation site at Ser^90^ in addition to those found on proteoform 2. The FAM20 strain can therefore harbor up to 4 different glycans on the same pilin monomer. This detailed top-down analysis of the mutant strain allowed us to precisely assign specific PTMs to the 23 different proteoforms found in the wild type FAM20 highlighting the tremendous diversity of structures found on the pilin of this strain (Fig 2).

**Table 1:** Measured neutral monoisotopic masses of PilE proteoforms from the FAM20*pglHpptA* double mutant and comparison with theoretical masses calculated from the FAM20 sequence plus indicated PTMs.

FAM20 *pglHpptA* proteoform	Measured monoisotopic neutral Mass (Da)	Theoretical mass (Da)	Error (ppm)	PTM present*
**1**	15693.0104	15692.8603	9.6	Methylated N-ter, C-C, 2 PG, 2 GATDH
**2**	15967.0251	15966.9768	3.0	Methylated N-ter, C-C, 2 PG, 3 GATDH
**3**	16241.1870	16241.0933	5.8	Methylated N-ter, C-C, 2 PG, 4 GATDH

* C-C, disulfide bond; PG, phosphoglycerol; GATDH, glyceramido trideoxyhexose

### The presence of multiple glycosylation sites is a common feature of strains expressing class II pilins

**Fig 3:**
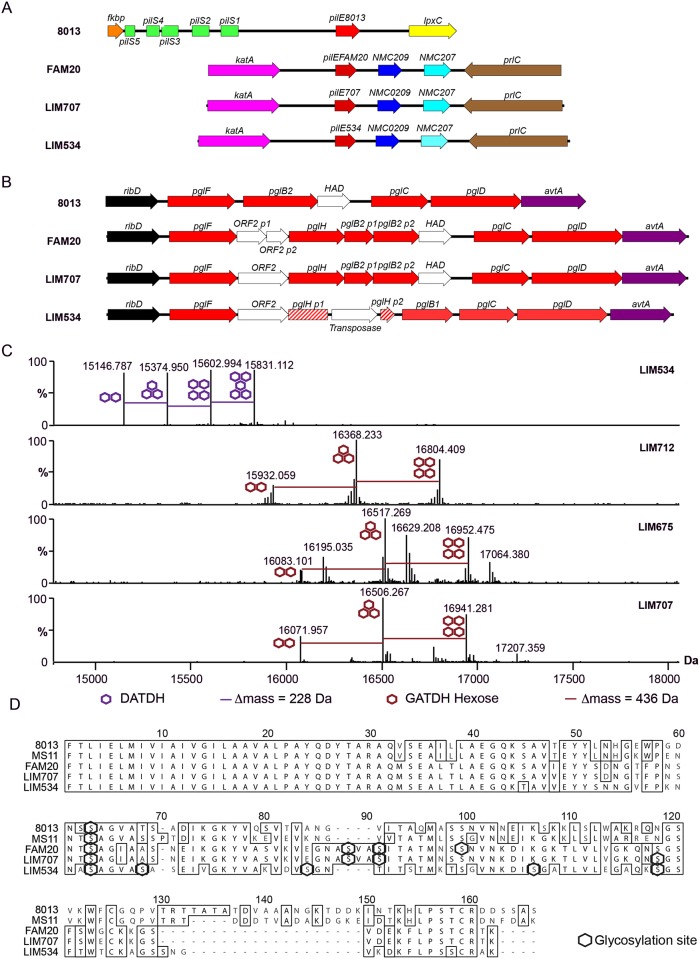
Genomic and biochemical description of class II pilin expressing strains isolated at different sites and at different times. (A) Genetic organization of the pilin locus in the 8013, FAM20, LIM707, and LIM534 strains. (B) Genetic organization of the core *pgl* locus in the same strains. (C) High resolution intact protein mass profiling of 4 class II pilin expressing strains demonstrating multisite glycosylation. (D) Alignment of the pilin sequences from collected class II pilin expressing clinical strains. Sequences of the *N*. *meningitidis* 8013 and FAM20 strains and the *N*. *gonorrhoeae* MS11 strain [35] are included for reference. Glycosylation sites irrespective of their nature appear as black hexagons.

The presence of a strikingly high number of pilin glycosylation sites in FAM20 raised the question of whether this is a particularity of FAM20 or a common feature of strains expressing class II pilins. We therefore collected several class II pilin expressing strains, isolated during a 2003–2006 period from patients suffering from sepsis and meningitis at the Limoges university hospital in France and analyzed their PTM (Table 2). Strains were selected to be part of different serogroups and sequence types to represent a diverse panel with the common feature of expressing class II pilins. To allow detailed genetic analysis the entire genomic sequence of two of these clinical strains was established and genes involved in pilin production and its glycosylation characterized (Fig 3A and 3B, LIM534 and LIM707). In both cases sequences of the major pilin are closely related to the FAM20 type II sequence (S2 Fig) and the pilin gene is also located between the *katA* and *prlC* genes as expected for class II expressing strains (Fig 3A). Type IV pili were purified from these strains and submitted to high resolution intact mass profiling. Overall, spectra were less complex than the FAM20 strain with 3–6 major proteoforms (Fig 3C). Nevertheless pilin purified from LIM534, LIM712, LIM675 and LIM707, consistently displayed evidence of multiple glycosylation sites (Fig 3C). The difference in mass between major proteoforms could be explained by the sequential addition of several DATDH/GATDH or DATDH-Hex/GATDH-Hex glycans depending on the strain. Top-down MS analysis of these different proteoforms demonstrated that proteoforms contained 2–5 glycosylation sites (Fig 3D). These results show that strains expressing class II pilins from different clonal complexes isolated from different continents and in different time periods share the common feature of carrying multiple glycosylation sites. This strongly suggests that such multisite pilin glycosylation is a general feature of class II expressing strains.

**Table 2:** Clinical isolates used in this study.

Strain	Isolation	Date	Pilin Class	CC*	Serogroup	Reference
**8013**	Blood	1989	I	ST-18	C	[58]
**FAM20**	CSF	1980s	II	ST-11	C	[26]
**LIM534**	Throat	2006	II	ST-5	A	[27]
**LIM712**	Blood	2005	II	ST- 11	C	This study
**LIM675**	Blood	2003	II	ST-11	C	This study
**LIM707**	CSF	2003	II	ST-11	C	This study

*CC, clonal complex

### The number of glycosylation sites is determined by the pilin primary structure

The observation that class II pilin-carrying strains bear multiple glycosylation sites as opposed to class I strains that carry only one, could be explained by two non-exclusive hypotheses. First, the particular primary structure of class II pilins may itself be more favorable to glycosylation due to a larger number of accessible serine residues. Second, this difference is due to the genetic background and in particular to the *pgl* genes expressed by these strains.

As a first attempt to address this question the genomic regions carrying the *pgl* genes were analyzed in two of the isolated class II pilin expressing strains, LIM534 and LIM707 but this did not reveal any obvious explanation for the number of glycosylation sites. For instance, the PglO/L oligosaccharide transferase was highly conserved between the class I (8013) and class II (FAM18, LIM534 and LIM707) pilin-expressing strains with identity scores between 98 and 100%. As in the class I pilin-expressing strains the *pglBCDFH* genes are localized between the *ribD* and *avtA* genes apart from the *pglA* and *pglE* genes which are located on a separate region (Fig 3B). The LIM707 strain carries a split *pglB2* gene (GATDH) previously found to maintain functionality and an insertion containing *orf2* and *pglH* between the *pglF* and *pglB* genes [20, 31]. The LIM534 strain expresses a *pglB1* (DATDH) gene and displays an insertion containing the *orf2* and *pglH* genes but, interestingly, the *pglH* gene is interrupted by a transposase explaining why only a monosaccharide is found on the pilin.

**Fig 4:**
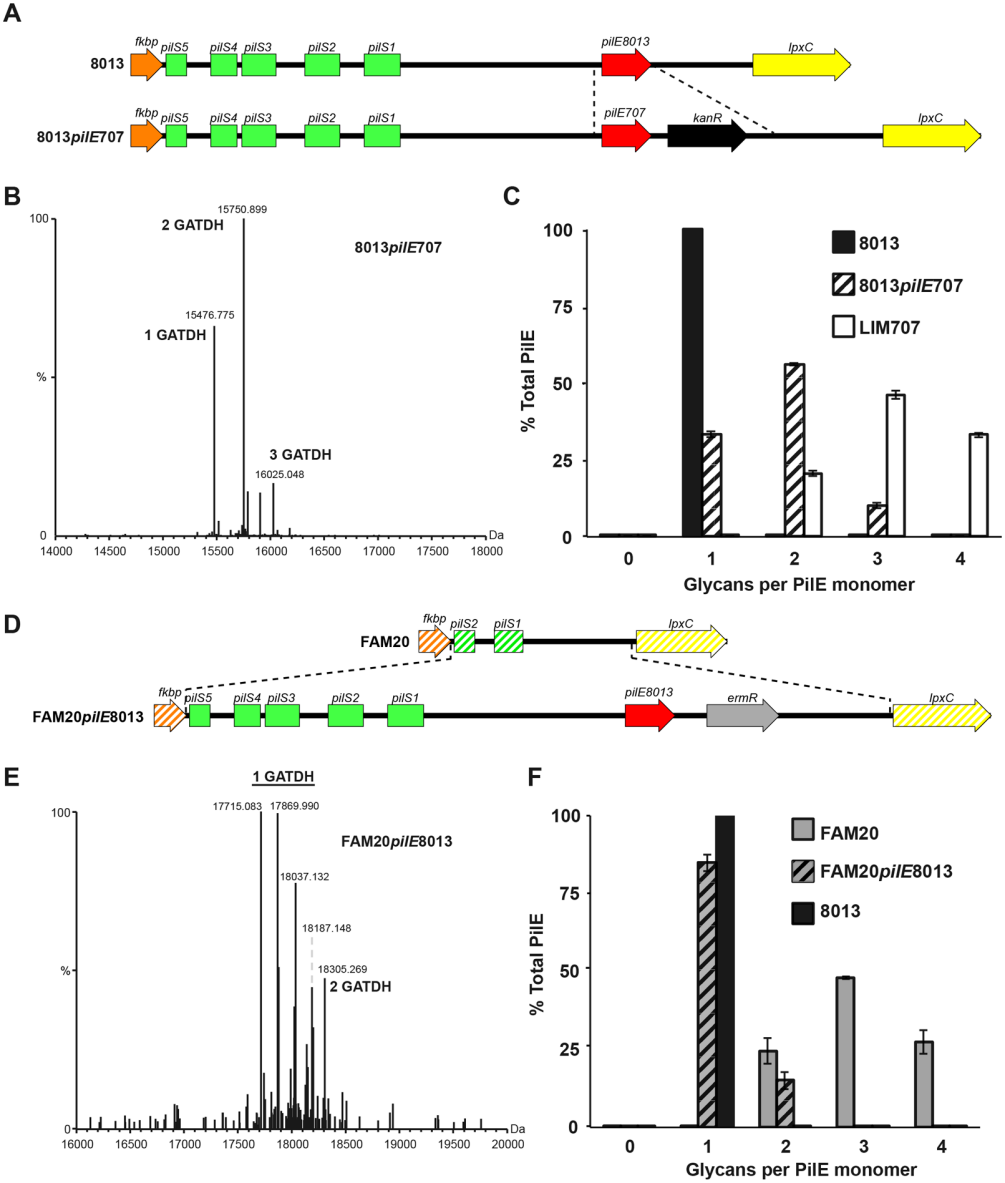
Roles of pilin amino-acid sequence and genetic environment in determining the number of glycosylation sites. (A) Schematic representation of the genetic modification introduced in the 8013 strain to express the class II pilin from the LIM707 strain (8013*pilE*707). (B) Whole protein mass spectrometry analysis of the 8013*pilE*707 strain. (C) Distribution of the number of glycans per monomer in the 8013, 8013*pilE*707 and LIM707 strains. (D) Schematic representation of the insertion introduced in the FAM20 strain to express the class I pilin from the 8013 strain (FAM20*pilE*8013). (E) Whole protein mass spectrometry analysis of the FAM20*pilE*8013 strain. In this case there are multiple proteoforms harboring one glycosylation site. This is due to different phosphoform content and the presence of either di or monosaccharide on Ser^63^. (F) Distribution of the number of glycans per monomer in the FAM20, FAM20*pilE*8013 and 8013 strains. For panels D and F, average and standard deviations are indicated from 3 independent pili preparations.

To address the potential role of the pilin sequence in determining the number of glycosylation sites we generated two “class swap” mutant strains, the first with a class II pilin in a class I pilin-expressing genetic background and the reciprocal strain with a class I pilin in a class II background. In the first case, a class II LIM707 pilin was expressed in the context of the 8013 background (8013*pilE*LIM707, Fig 4A). Pilin from the 8013 strain normally harbors GATDH at a single glycosylation site [18]. Expression of the class II LIM707 pilin in the 8013 background strain led to a pilin modified with up to 3 GATDH moieties (Fig 4B and 4C). The glycosylation sites were the same as in the original LIM707 strain. This result indicates that the *pgl* genes from the 8013 strain are capable of modifying the pilin at multiple sites and that the number of glycosylation sites is determined by the pilin sequence itself rather than the *pgl* genes. To confirm this result the reverse situation was generated and the 8013 pilin (class I) was expressed in the FAM20 background (FAM20*pilE*8013, Fig 4D). High resolution MS analysis showed that pilins purified from this strain comprised of a more complex array of proteoforms due to modification with PE, PC, di and trisaccharides but the vast majority contained a single glycosylation site at Ser^63^ (Fig 4E and 4F). Interestingly, in this case about 10% of the pilin also contained a second glycosylation. Taken together these results show that the genetic environment of the strain determines the type of sugar added, DATDH or GATDH, mono, di or trisaccharide but the presence of multiple glycosylation sites in class II pilins is largely determined by the primary structure of this class of pilins.

### Multiple glycosylation sites on class II pilins affect type IV function in a strain-specific fashion

*Neisseria meningitidis* class I pilin glycosylation has been shown to contribute to adhesion by interacting with the platelet activating factor (PAF) receptor on the surface of human airway cells [32]. In contrast, in *Neisseria gonorrhoeae* strains deficient for pilin glycosylation exhibited an early hyper-adhesive phenotype but were attenuated in their ability to invade primary cervical epithelial cells [33]. The multiple glycosylation sites found on the class II pilins raised the question of their function more acutely. To explore the function of glycosylation FAM20, LIM707, LIM534 and 8013 strains deleted for the *pglC* and *pglD* genes were generated. Surprisingly, however, despite repeated attempts we were unable to purify pili from the FAM20*pglC* and FAM20*pglD* strains. Electron microscopy observation showed that these two strains do not display any type IV pili on their surface (S2 Fig). Complementation of mutant strains with the corresponding genes restored piliation. In the case of the FAM20 strain, glycosylation appears to be necessary for efficient pilus assembly. This result was unique to the FAM20 strain as the other two class II pilin expressing strains showed normal piliation in absence of glycosylation.

**Fig 5:**
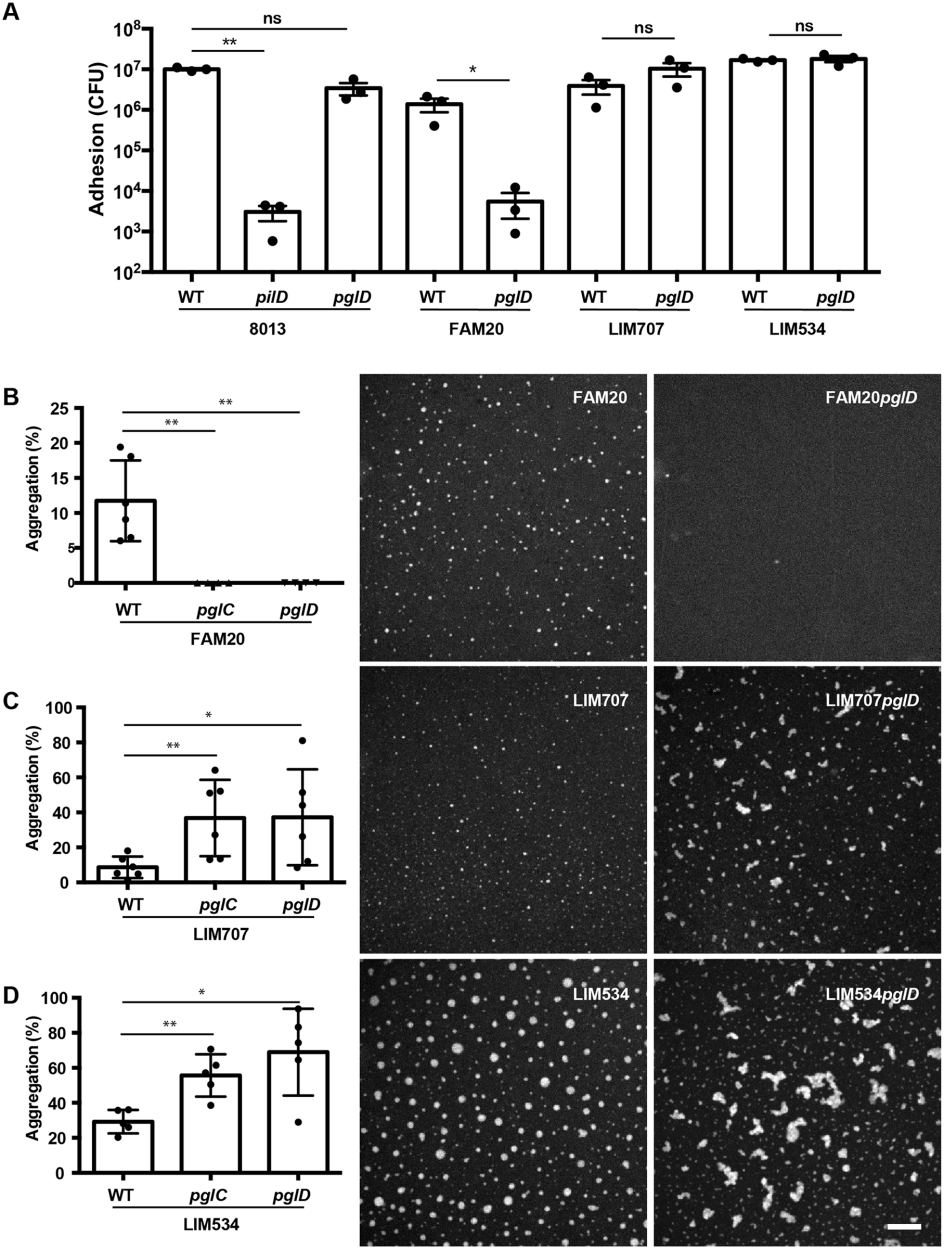
Functional consequences of absence of glycosylation in class II strains. (A) Impact of class II pilin glycosylation on adhesion to endothelial cells. Strains 8013, LIM707, LIM534, FAM20 and their corresponding *pglD* mutants were allowed to adhere to endothelial cells for 4 hours and the number of adherent bacteria analyzed. A non-piliated *pilD* mutant of the 8013 strain was used as a negative control. (B-D) Quantitative and qualitative impact of pilin glycosylation on bacterial aggregation in three class II pilin-harboring strains: (B) FAM20, (C) LIM707 and (D) LIM534. Bacteria were allowed to aggregate in suspension and the size and number of aggregates determined using fluorescence microscopy following DAPI staining. The scale bar indicates 200 μm. Mean ± standard deviation was determined and indicated on the figures. Statistical analysis was done by paired two-tailed t-test; * indicates P ≤ 0.05; ** indicates P ≤ 0.01.

The impact of the loss of glycosylation on the typical pilus properties of adhesion to endothelial cells and auto-aggregation was then determined (Fig 5A–5D). As expected from the absence of pili, the FAM20*pglD* strain showed very low adhesive capacity indistinguishable from the non-piliated mutant (Fig 5A). In contrast, adhesion by the LIM707, LIM534 and 8013 strains were unaffected by the absence of pilin glycosylation. Similar results were found on pulmonary epithelial cells (S3 Fig). Bacterial aggregation was evaluated as a second type IV pilus-dependent property (Fig 5B–5D). As expected, the FAM20*pglC* and *pglD* mutants did not show any aggregation. Bacterial aggregation of LIM534 and LIM707 was higher in the absence of glycosylation. Interestingly, instead of being spherical, bacterial aggregates formed by the unglycosylated LIM707 and LIM534 strains displayed unusual heterogeneous shapes. Such an aggregation phenotype characterized by more aggregation and polymorphous aggregates is reminiscent of strains deficient for the PilT retraction ATPase [34]. To evaluate whether the absence of glycosylation was altering pilus retraction the motility of these strains was evaluated (S4 Fig). Twitching motility depends on cycles of pilus extension and retraction that drag the bacteria on a surface. The glycosylation mutants of the LIM534 and LIM707 strains did not show any defect in motility indicating normal retraction on individual bacteria (S4 Fig). This suggests that class II pilin glycosylation destabilizes pilus-pilus contacts allowing for dynamic interactions between pili in the context of aggregates.

Taken together these results show that the absence of glycosylation on class II pilins leads to strong functional changes, and such changes vary depending on the strain. In the most dramatic situation pili were not expressed on the surface. In other cases, pilus-pilus interactions were stabilized leading to enhanced aggregation.

### Modeling pili structures with multiple glycosylations reveals extensive pilus surface coverage

**Fig 6:**
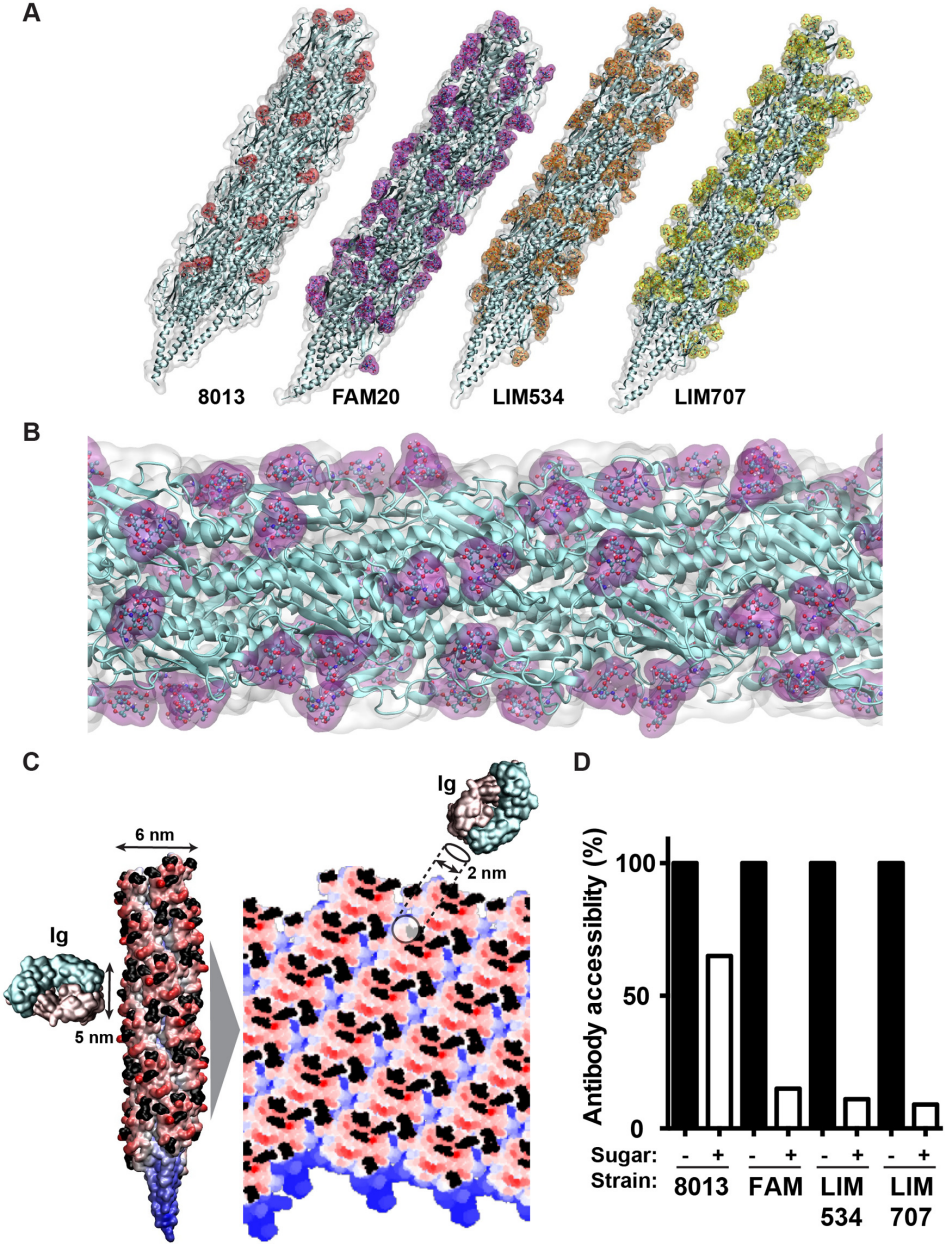
Molecular modeling of antibody accessibility to the primary structure of class II pilins. (A) Overall aspect of the glycosylated pilus fibers from the 8013, FAM20, LIM534 and LIM707 strains. The polypeptide structure appears in cyan and the surface representation of the sugars appears in color. (B) Detailed view of the FAM20 fiber formed with the proteoform with three glycosylation sites. Sugars appear in purple. (C) Projection of the pilus surface on a 2D grid. The antigen-binding tip of an antibody is represented at the same scale. Sugar moieties appear in black. (D) Antibody accessibility to the polypeptide chain in the presence or absence of sugar modifications. Results are presented as the percentage of positions along the grid that allow a 2 nm disc to be placed on the pilus surface without touching sugar moieties relative to all positions on the grid.

The important functional impact of the multisite glycosylation displayed by class II pilins described above suggests that sugars occupy a large percentage of the pilus surface. To explore this hypothesis the structures of pilin fibers were modeled using the *N*. *gonorrhoeae* MS11 pilin as a template [35] and taking into account glycan PTM. Three glycans per monomer were included in the model as it represents the dominant and average proteoform. Pilus assembly was performed as previously described [36] and corresponding sugars were built by energy minimization and added onto the pilus fiber. Organization of the whole structure was then refined first *in vacuo* and then in water. Glycosylated pilus structures formed of class II pilins consistently show global coverage of the fibers by sugars (Fig 6A). Higher magnification of the FAM20 pilus glycosylated on 3 sites per monomer, the most abundant proteoform, shows extensive coverage of the pilus surface (Fig 6B). Glycosylation therefore strongly changes the structure of the pilus fiber.

These results also suggest that glycosylation will perturb antibody recognition of the pilus fibers. In particular, antibodies directed against the pilus structures would have limited direct access to the protein backbone. As this could compensate for the absence of sequence variation in the class II pilins we decided to investigate this point further. All atoms of the pilus in cylindric coordinates were projected on a plane according to their height and angular coordinate, in order to obtain a flat representation of the pilus surface on a 2D grid (Fig 6C). The surface at the tip of antibodies, typically involved in antigen binding, is roughly circular in shape with a diameter in the order of 5 nm. Since interaction with the antigen does not require the whole surface we approximated the antibody-antigen binding site by using a 2 nm diameter disc [37]. The disc was tested against each position along a grid covering the pilus surface and for each position the presence of sugar was evaluated. The percentage of positions where antibody binding was not affected by sugars was then determined (Fig 6D). For the 8013 class I expressing strain that displays a single glycosylation site the presence of sugars decreased antibody accessibility to 65% of the surface. In the case of the class II pilins that carry multiple glycosylation sites, antibody accessibility was reduced to 15%, 11% and 9% of the surface in the FAM20, LIM534 and LIM707 strains respectively. These results show that the surface-accessible amino-acid residues of class II pilins are largely masked by glycosylation sites.

## Discussion

Our results show that, unlike in class I pilins, a large portion of the pilus surface is coated with sugars in class II pilins. Over the years pilin glycosylation of class I pilins has been studied from 3 different *Neisseria meningitidis* strains demonstrating a single conserved glycosylation site at Ser^63^ (Table 3). Strain C311#3 displays a Gal(β1–4)Gal(α1–3)2,4-DATDH [16], strain 8013 one GATDH residue [14] and NIID280 a DATDH residue [17]. In addition, *N*. *gonorrhoeae* strain N400 presents a hexose residue linked to a DATDH on its class I pilin also at Ser^63^ [15]. In this single study using top-down mass spectrometry, we describe for the first time the glycosylation pattern of 5 different strains expressing class II pilins including the FAM18 reference strain. Pilins from all of these 5 strains display 3 to 5 glycosylation sites (Table 3). Independently of the serogroup, clonal complex, geographic site or temporal period of isolation of the strains (Table 2), class I pilins show a single site of glycosylation while class II pilins have multiple sites of glycosylation.

**Table 3:** Summary of the number of glycosylation sites found in the different *Neisseria meningitidis* strains.

Strain	Pilin class	Number of sites	Reference
**C311**	I	1	[16]
**8013**	I	1	[14]
**NIID280**	I	1	[17]
**N400**	I	1	[15]
**FAM20**	II	4	This study
**LIM534**	II	5	This study
**LIM712**	II	4	This study
**LIM675**	II	4	This study
**LIM707**	II	4	This study

Molecular modeling reveals that multisite glycosylation of the pilin monomer leads to the coverage of the pilus surface. This could have important consequences in terms of adaptation of the bacteria to the host immune response. More specifically, this could explain why amino-acid sequence variation is not required in class II strains because the polypeptide chain is not exposed to the extracellular milieu and thus not submitted to pressure by the immune system. That is not to say that antibodies cannot recognize glycosylated class II pilins: indeed glycopeptides from type IV pili are immunogenic [38, 39]. Rather, these results suggest that the immune escape scenario would then be different between class I and II pilins. In the case of class I pilins, after throat colonization by a given strain the IgAs specific for the pilin primary sequence will be produced and lead to killing of the initial strain, but variants arising from recombination at the pilin genetic locus will survive until a second adaptation of the immune system. This cycle can potentially repeat itself numerous times. In the case of class II strains the primary structure is cloaked in oligosaccharides, and only antibodies targeted to epitopes that include sugar moieties will efficiently lead to bacterial killing. In this case variants in the sugar structure will survive. Type IV pili have been considered as potential vaccine antigens against *Neisseria gonorrhoeae* infections but sequence variation in class I pilins has hampered these attempts [40, 41]. In the absence of sequence variation in class II pilins it could be tempting to use such proteins as vaccine antigens but our results show that glycosylation would complicate this approach.

A number of arguments support the idea that sugar structure does change during infection of individuals and during epidemics. It has been shown that sera from infected patients during acute and convalescent stage meningococcal disease recognize the major pilin [9] and thus establishes that type IV pili are indeed a target of the immune system and its pressure. It is also well documented that certain *pgl* genes such as *pglA* and *pglE* are submitted to phase variation [42]. This implies that the functionality of these genes and thus the nature of pilin glycosylation can vary at rates between 10^−2^ to 10^−6^ per cell per generation giving the bacteria the opportunity to evade antibody response against type IV pili [43, 44]. Beyond phase variation, *pgl* genes appear to be the site of rapid changes including at the epidemic scale. Lamelas *et al*. performed a longitudinal study in Northern Ghana between 2001 and 2009 [45] where they collected and sequenced the genomes of 100 strains in order to identify evolutionary changes during these epidemic waves. This revealed that the *pgl* genes were the site of no less than 5 successive recombination events during this period. Importantly, all of the strains in this study display class II pilins (Gerd Pluschke, personal communication). Our work also provides evidence of changes in pilin glycosylation. In the case of the LIM534 strain the *pglH* gene is interrupted by an insertion sequence. This insertion event is a molecular signature of changes in the nature of the sugars coating a class II pilin. Taken together these studies underline the high level of variation undergone by class II pilin glycosylation likely to evade the immune response directed against pili.

Our results also provide an explanation for the molecular mechanism that leads to multisite glycosylation in class II pilins. We showed that expression of a class II pilin in a strain normally expressing a class I pilin leads to glycosylation on multiple sites on the pilin backbone. This result shows that the pilin amino-acid sequence is a determining factor for multisite glycosylation. Alignment of the pilin sequences from class I and II strains suggests two scenarios. Certain glycosylated serines present on class II pilins are simply absent in class I pilins (e.g. serines at alignment positions 88, 91 or 118 in class II are absent in class I). Alternatively the serine is present on class I pilins but the local sequence is different (e.g. serines at alignment position 68 and 99) and this would likely affect glycosyltransferase recognition. Predominance of pilin sequence in the determination of glycosylation sites is confirmed by the reciprocal situation. When a class I sequence is expressed in the class II expressing background, the vast majority of the pilin contains only one sugar. It is noteworthy however that the genetic background, and most probably the *pgl* genes, also plays a partial role in determining the number of glycosylation sites. Indeed, when the class II pilin is expressed in its normal background the main proteoform contains 3 glycans per monomer whereas when it is expressed in the class I expressing background the main proteoform contains only 2. Furthermore, when the class I sequence is expressed in the class II expressing background the main proteoform contains one sugar but 10% of pilins also contain 2 sugars. It is therefore likely that pilins of different classes have co-evolved with their respective *pgl* systems and the glycosylation systems in class II pilin expressing strains are more efficient.

Independently of the number of glycosylation sites, the nature of the sugars on the pilin is determined by the *pgl* genes. The FAM20 strain expresses a *pglB2* allele, *pglA* and *pglE* genes are in the OFF phase and the insertion with the ORF2 and *pglH* genes is present. Accordingly, pilins from this strain are modified with a GATDH core, determined by the *pglB2* allele, and between 1 and 2 hexose residues likely being a glucose transferred by PglH. Interestingly, the PglH transferase expressed by this strain is partially functional in the sense that certain sites display a GATDH-hexose while others a GATDH monosaccharide. This specificity of the *pglH* allele contributes to the complexity of the pattern found on pilins expressed by the FAM20 strain. The LIM707 strain contains the same expression pattern of *pgl* genes as the FAM20 (*pglB2* allele, *pglA*_*OFF*_, *pglE*_*OFF*_, *pglH* present) leading to the production of a GATDH-Hexose type of sugar. In contrast to the FAM20 strain, all sugars on the LIM707 pilin are disaccharides. For the LIM534 strain, we observe the *pglB1* allele, *pglA* and *pglE* alleles are in the OFF phase and the *pglH* gene is present but interrupted by an insertion sequence. In this case, only DATDH is present as predicted by the genomic data. When pilin sequences are introduced into a different genetic background the nature of sugar changes with the genetic background of the strain. When the pilin gene from the LIM707 strain is introduced in the 8013 background, the pilin becomes modified with a GATDH residue as found in the 8013 strain rather than with a GATDH-Hexose disaccharide. Similarly, introduction of the 8013 pilin gene into the FAM20 strain leads to the expression of pilin modified with GATDH-hexose. Overall these results show that the pilin sequence determines the *number* of glycosylation sites and the *pgl* gene pattern the *nature* of the sugar.

An intriguing result of this study is the difference in functional consequence of the lack of glycosylation in the different class II pilin expressing strains. The most striking phenotype is in the FAM20 strain where type IV pili are simply not expressed on the surface of the bacteria in absence of glycosylation. This phenotype is specific to the FAM20 strain as the LIM707 or LIM534 strains still adhere to host cells via their type IV pili despite inactivation of the *pgl* genes. Another specificity of the FAM20 strain is the high number of proteoforms expressed. In addition to the 2–4 glycosylation sites displayed by this strain, phosphoglycerol (2) phosphoethanolamine (1–3) and phosphocholine (2) modifications are also present. It is possible that in absence of sugar these numerous modifications generate a specific structural environment that is incompatible with pilus expression. It is also possible that the piliation machinery has co-evolved with the glycosylation of this strain and that specific interactions with the machinery such as with the PilQ secretin require glycosylation. Further work is required to elucidate at which step glycosylation is necessary for piliation in the FAM20 strain. For instance, identification of the point at which pilus biogenesis is blocked in the FAM20*pglC/D* strains will yield useful information to understand the mechanisms of pilus biogenesis. A role for glycosylation in the assembly and function of pili in other organisms has been described. In *Neisseria gonorrhoeae*, pilin (class I) glycosylation has subtle effects on pilus dynamics [46]. Perhaps a similar but more prevalent mechanism is at play in the FAM20 strain. Glycosylation of the *Pseudomonas aeruginosa* major pilin is also necessary for efficient piliation [47]. In Archea, the archaellum, a swimming organelle closely related to type IV pili bears glycosylation sites that are required for assembly [48]

Surprisingly, in strains LIM707 and LIM534 absence of glycosylation of their class II pilins did not affect adhesion to either endothelial or epithelial cells. This is in contrast with previous studies on strains expressing class I pillins that reported a decrease of adhesion in strains lacking pilin glycosylation due to direct interactions of the sugars with cellular receptors [33]. Recently, the recombinant non-glycosylated class I pilin from the 8013 strain was shown to interact with the surface protein CD147 [49]. In principle class II pilins could mediate interactions with cellular receptors through the polypeptidic chain but modeling shows that its accessibility is limited by the numerous surface exposed glycans. Intriguingly, our results thus make a direct interaction between the major pilin and a cellular receptor difficult to imagine at the structural level. Further structural work is required to clarify this point. In contrast, the absence of glycosylation of the pilins expressed by LIM707 and LIM534 led to enhanced aggregation and to an unusual aggregation behavior likely due to stabilized pilus-pilus interactions. These results are consistent with the idea that multisite class II pilin glycosylation leads to changes in surface properties of type IV pili. These results also point out that the functional consequences of pilin glycosylation could be different in class I and class II pilins.

Overall, our work revises the current view of pilin glycosylation. Starting from a single modification per pilin in class I strains we now realize that a large proportion of *N*. *meningitidis* strains express class II pilins and carry multiple glycosylation sites. Our study first reveals the profound implications in terms of pilus biogenesis, structure and function. The results presented here also have important implications in terms of immunity against type IV pili, vaccine design and how *N*. *meningitidis* manages to escape the immune system. In particular the presence of multiple glycosylation sites provides a simple explanation for the absence of pilin sequence variation in class II pilins and suggests that variations in sugar structure are the main motor for immune evasion in these strains. The worldwide distribution, hypervirulence and association with epidemic forms of the disease of strains carrying class II pilins underscore the importance of these results to understand *Neisseria meningitidis* infections. In the more global context of infectious diseases our study highlights the wealth of strategies exploited by pathogens to escape the immune system and the key role played by glycosylation.

## Materials and Methods

### Bacterial strains and growth conditions

*N*. *meningitidis* strains were grown on GC agar base plates (Conda Laboratorios, Spain) containing Kellogg's supplements [7] and, when required, 5 μg/ml chloramphenicol at 37°C in moist atmosphere containing 5% CO_2_. *Escherichia coli* transformants were grown in liquid or solid Luria-Bertani medium (Difco) containing 100 μg/ml ampicillin. *Neisseria meningitidis* strains used in this study are described in Table 4.

**Table 4:** Genetically modified strains used in this study.

Strain	Genotype	Reference
**8013*pglC***	8013*ΔpglC*::*mariner*	[59]
**8013*pglD***	8013*ΔpglD*::*mariner*	[59]
**FAM20*pglH***	FAM20*ΔpglH*::*alphA-3*	This study
**FAM20*pptA***	FAM20*ΔpptA*::*mariner*	This study
**FAM20*pglHpptA***	FAM20*ΔpglH*::*cat ΔpptA*::*mariner*	This study
**FAM20*pglC***	FAM20*ΔpglC*::*mariner*	This study
**FAM20*pglD***	FAM20*ΔpglD*::*mariner*	This study
**LIM707*pglC***	LIM707*ΔpglC*::*mariner*	This study
**LIM707*pglD***	LIM707*ΔpglD*::*mariner*	This study
**LIM534*pglC***	LIM534*ΔpglC*::*mariner*	This study
**LIM534*pglD***	LIM534*ΔpglD*::*mariner*	This study
**FAM20*pilE*8013**	FAM20 *ΔpilE*::*pilE8013*	This study
**8013*pilE*707**	8013 *ΔpilE*::*pilE707*	This study

### Molecular biology techniques

#### Genomic sequencing of clinical strains

Genomic sequencing of the LIM534 and LIM707 strains was performed with an Illumina sequencer with 2x74 paired-end sequences. Sequencing was performed at the sequencing platform IMAGIF (Centre de Recherche de Gif– www.imagif.CNRS.fr). Sequences were then assembled with the CLC Genomics Workbench 7 software using the assembly tool and submitted to the BIGSdb (http://pubmlst.org/software/database/bigsdb/).

#### Gene inactivation

Mutations in the *pptB*, *pptA* and *pglD* genes were described elsewhere [36]. To delete the *pglH* gene, an upstream region amplified with PglH-NF and PglH-NR primers (Table 5) and a downstream region amplified with PglH-CF and PglH-CR primers were restricted by the corresponding enzymes and ligated in the pBluescript plasmid restricted with SalI and SacI (Stratagene), then the kanamycin resistance cassette was cloned in the BamHI site.

**Table 5:** Oligonucleotides used in this study.

Primer	Sequence*	Enz.
***pglH*NF2**	**GTCGAC**GGAATTTCATTTCCGGAAAAC	*SalI*
***pglH*NR**	**GGATCC**AGCAATAAGGGGCGACGATG	*BamHI*
***pglH*CF**	**GGATCC**AAGTTGCCGAAGTCCTTAC	*BamHI*
***pglH*CR**	**GAGCTC**ACCGCCAGATTGAAAATGC	*SacI*
**E707F**	GAGGCATTTCCTTTCCAATTAGGAGTAATTTTATGAAAGCAATCCAAAAAGGTTTC	
**E707R**	CCATTTATTATTTCCTTCCTCTTTTCTGATCCTTACTTATTTGGTGCGGCAGGTAGA	
**8013-lpxC-pilE_FWD**	GCCGTCTGAAGCGTCGGGCAAATCATCGC	
**8013-lpxC-pilE_REV**	GATTGTGATTGCCATCGTCG	
**FAM20-pilE_FWD**	gccgtctgaaATTTGGTGCGGCAGGTAG	
**FAM20-pilE_REV**	AGGTTTCACCCTGATCGAG	
**McPilRBS**	gcatttcctttccaattaggag	
**3-end-ErmR_FWD**	cgttatgaaatgggttaaca	
**Mid_pilEandS1FWD**	GTCAGCAGTGCCGAAAATTGTCAG	
**Late_pilEandS1FWD**	CCTCGTCGGTGCAGAAACTTA	
**3’end_pilS1 FWD**	ctcggtgacggctgatttttgac	
**5’END_FKBP REV**	gctgaaagtgtacgaataaAGC	
**3’END_PILS5 REV**	CTGACATAAtggcttcaag	
**3END8013REVPILS1**	CATCCTTTTGGTCgaaggtc	
**3END8013FORPILS1**	gaccttcGACCAAAAGGATG	
**3END8013REVPILS2**	GACGAAGCTATCCTTttggccg	
**428BP_PRE-PILS1 FOR**	CATCGGTACGGAAACTTATCG	
**FAM18-PILE_FWD**	gccgtctgaaATTTGGTGCGGCAGGTAG	
**FAM18-PILE_REV**	AGGTTTCACCCTGATCGAG	

* Restriction sites in bold

#### Allelic exchange

The megaprimer strategy [50] was used to substitute the type II pilin sequence from the LIM707 strain into the 8013 strain normally expressing a class I pilin (8013*pilE*707). The first amplification was done using E707-F and E707-R primers using genomic DNA from the LIM707 strain as a template. The amplicon was used as a megaprimer to amplify the TopoPCR2.1 vector containing the 8013 pilin sequence followed by a kanamicyn resistance cassette [51]. Amplification products were treated with DpnI and transformed into *E*. *coli*. Plasmids from resulting clones were sequenced to identify those carrying the LIM707 pilin sequence. A positive plasmid was selected for transformation into the 8013 strain.

To introduce the 8013 *pilE* and *pilS* loci into FAM20, first the *lxpC* to *pilE* fragment was amplified from 8013 chromosomal DNA using the primers 8013-lpxC-pilE_FWD and 8013-lpxC-pilE_REV. and cloned into the plasmid pBlunt (Life Technologies). The *ermC* resistance gene was cloned downstream of *pilE* into the PmeI site. This construct was transformed into 8013 selecting for ErmR and the adjacent DNA sequenced. Chromosomal DNA from the ErmR 8013 was used to transform FAM20 selecting for ErmR, and transformants were screened by PCR using primers McPilRBS and3-end-ErmR_FWD to identify transformants that had recombined the *pilE* gene along with *ermC*. These transformants were further analyzed using primers Mid_pilEandS1FWD, Late_pilEandS1FWD, 3’end_pilS1 FWD, 5’end_fkbP REV, 3’end_pilS5 REV, 3end8013REVpilS1, 3end8013FORpilS1, 3end8013REVpilS2, and 428bp_pre-pilS1 and long PCR conditions (LongAmp Taq, NEB) to identify transformants carrying the entire *pilS* locus from 8013. DNA sequence analysis of PCR products was performed to confirm the presence of all 8013 sequences between *lpxC* and *fkbP* in FAM20.

The FAM20 native, class II *pilE locus* was cloned by amplifying the FAM20 *pilE* using primers FAM20-pilE_FWD and FAM18-pilE_REV and cloning into pSMART HC Amp (Lucigen). After confirmation by sequence analysis, plasmid DNA was treated with EZ-Tn5<KAN-2> (Epicenter) and the mini-transposon insertion site identified by PCR as 95 bp downstream of the translation start site. This construct was then transformed in the FAM20 strain to interrupt the endogenous *pilE* gene and to generate the FAM20*pilE*8013 strain.

### Mass spectrometry techniques

#### PilE preparation

Pili were prepared as described previously [36]. Briefly, bacteria from 10–12 Petri dishes were harvested in 5 mL of 150 mM ethanolamine at pH 10.5. Pili were sheared by vortexing for 1 min. Bacteria were centrifuged at 4,000xg for 30 min at 4°C and the resulting supernatant further centrifuged at 15,000 x g, 30 min, ambient temperature. The supernatant was removed, pili precipitated from the suspension by the addition of 10% (vol/vol) ammonium sulfate saturated in 150 mM ethanolamine pH 10.5 and allowed to stand for 1 h. The precipitate was pelleted by centrifugation at 4,000xg for 1 h at 20°C. Pellets were washed twice with PBS and suspended in 100 μL distilled water.

#### High resolution mass profiling

Protein samples were desalted by C_4_ ZipTip (Millipore) and eluted directly into a 10 μL spray solution of methanol:water:formic acid (75:25:3). A small amount, 2–6 μL, was introduced into either an Orbitrap Velos mass spectrometer, equipped with ETD module (Thermo Fisher Scientific, Bremen, Germany) or Orbitrap Fusion mass spectrometer (Thermo Scientific, San Jose CA) using a TriVersa NanoMate (Advion Biosciences, Ithaca, NY, USA) in positive ion mode. The spray voltage was set to 1.2–1.6 kV and back-pressure to 0.3–0.4 psi. A full set of automated positive ion calibrations was performed immediately prior to mass measurement.

For Orbitrap Velos MS the transfer capillary temperature was lowered to 100°C, sheath and auxiliary gasses switched off and source transfer parameters optimised using the auto tune feature. Helium was used as the collision gas in the linear ion trap. The FT automatic gain control (AGC) was set at 1x10^6^ for MS experiments. Spectra were acquired in the FTMS in full profile mode with between 1 and 20 microscans over several minutes, with averaging on and set to the maximum value, and a resolution of 60,000 at *m/z* 400. The final few spectra were then averaged using Qualbrowser in Thermo Xcalibur 2.1 and deconvoluted using Xtract to produce zero charge mass spectra.

For Orbitrap Fusion MS transfer capillary temperature was lowered to 100°C, sheath and auxiliary gasses switched off and protein mode switched on. The pressure in the ion routing multiple was lowered to 4 mTorr and ions detected directly in the Orbitrap. Spectra were acquired at resolutions of 120,000 at *m/z* 200 with between 1 and 20 microscans over several minutes with averaging. Processing was performed as for spectra acquired with the Orbitrap Velos.

#### Top-down mass spectrometry & data analysis

For top-down MS/MS experiments performed on the Orbitrap Velos the FT automatic gain control (AGC) was set at 2x10^5^. Ions corresponding to the isotopic distribution of a single charge state were selected with the largest possible window to avoid overlap with neighbouring species but minimize signal loss. ETD was performed using fluoranthene as the reagent gas. Interaction times were varied to maximise sequence coverage but were kept below 20 ms. Supplemental activation was used as noted. Spectra were acquired in the FTMS in full profile mode at a resolution of 60,000 at m/z 400, with between 10 and 50 microscans and with averaging on and set to the maximum value. The final few spectra were then averaged using Qualbrowser in Thermo Xcalibur 2.1 and deconvoluted using Xtract to produce singly charged MS/MS spectra.

Top-down experiments on the Orbitrap Fusion were done in a similar way with ion selection performed in the quadrupole, ETD performed in the linear ion trap at interaction times of 3–10 ms and fragment ions detected in the Orbitrap at high resolution.

Peak list data resulting from the deconvolution of several spectra (often different charge states of the same species or ETD experiments performed on the same charge state but with different interaction times) were combined and imported into a home built package for ion assignment and automated fragmentation map creation. Low mass, low charge ions that are often clearly present in the spectra but missed by the Xtract algorithm were added manually to the list and PTM assignment was performed with this software tool.

### Functional assays

#### Bacterial aggregation assay

Bacteria grown on GCB agar plates were adjusted to OD_600_ of 0.05 and incubated for 2 hours at 37°C in RPMI containing 10% FBS. The bacterial suspension was concentrated to OD_600_ of 0.3 by centrifugation at 15000 x g for 1 min followed by resuspension in medium containing DAPI (4’, 6- diamidino-2-phenylindol; 0.1 μg/ml). Bacterial suspensions were briefly vortexed and transferred in a glass-bottom 96-well plate (Ibidi GmbH, München, Germany). After a 30 min incubation step, aggregates were observed microscopically with a 4x lens and size and numbers of bacteria involved in aggregates relative to the total amount of bacteria were determined with the ImageJ software, as previously described [36].

#### Bacterial adhesion assay

Adhesion of meningococci to human umbilical vein endothelial cells (HUVECs) was done as described previously [52]. Cells were grown at 37°C in a humidified incubator under 5% CO2. HUVECs (Promo- Cell, Heidelberg, Germany) were used between passages 1 and 8 and grown in Endo-SFM (Gibco) supplemented with 10% heat-inactivated fetal bovine serum (FBS, PAA Laboratories GmbH, Pasching, Austria) and endothelial cell growth supplement (Harbor Bioproducts, Norwood, USA). Monolayers of 10^5^ cells in 24-well plates were infected with 10^7^ bacteria (MOI of 100). The inoculum was characterized by CFU counts. After 30 min, unbound bacteria were removed by three washes and the infection was continued for 4h. Finally, after 3 washes adherent bacteria were recovered by scraping the wells and counted by plating appropriate dilutions on GCB agar plates.

Adhesion of meningococci to the human epithelial cell line A549 was performed as described for HUVECs except that a MOI of 500 was used instead of 100. A459 cells were a gift from Prof. Claire Poyart (Institut Cochin, Paris), cultured in DMEM high glucose, GlutaMAX, pyruvate (Life-Technologies) supplemented with 10% heat-inactivated FBS and maintained at 37°C and 5%CO2 in a humidified incubator.

#### Twitching motility assay

Twitching motility assays of *N*.*meningitidis* were performed inside a flow chamber (Ibidi GmbH, München, Germany). Bacteria (2.5x10^7^) were introduced into the flow chamber and incubated for 30 min at 37°C. Unbound bacteria were removed by 3 washes. Bacterial motility was monitored by video microscopy over a 2-minute period with the acquisition of 2 frames per second. Cell tracking was then analyzed with the ImageJ software using the spot tracking plug-in (http://icy.bioimageanalysis.org). Velocities of single bacterial tracks were calculated in time intervals of 2 s.

### Molecular modeling

#### Homology modeling

Target sequences were aligned to the MS11 *N*. *gonorrhoeae* pilin sequence, which shares high sequence identity with our strains: 77%, 56%, 59% and 59% for 8013, LIM534, LIM707 and FAM20, respectively. Individual pilin were modeled with Modeller 9v8 [53], using unmodified amino-acids sequences. The *N*. *gonorrhoeae* pilin structure (PDB code 2HIL) was used as template. 500 homology models were built with the standard procedure, clustered with the MMTSB Tool Set (www.mmtsb.org), and the best structure was selected according to the Dope assessment score [54], in the most populated cluster.

#### Pilus modeling

The approach for building the pilus was adapted from the multi-stage procedure described by Chamot-Rooke *et al*. [36]. We used CNS [55] with a modified version of the CHARMM19 force field, which included hand-made topology and parameters for the glycosylated sites (DATDH, GATDH, GATDH-Hex). The helical properties of the pili were taken from the *N*. *Gonorrhoeae* structure (rise 10.5 Å, angle 105.5°) and enforced throughout the modeling using the NCS STRICT command in CNS. In this way, only one unit is explicitly modeled with 20 virtual neighbors. First, we built the structures of the glycosylated sites by quick minimization with a simplified non-bonded interaction (repulsive Van der Waals only). The rest of the pilin was kept rigid during this stage. The second stage was a refinement *in vacuo* of the whole pilin, using adapted non-bonded parameters [36]. The third stage was a refinement in water, similar to the one used in NMR structure determination [56]. During the second and the third stage, the initial structures were maintained in a flexible and adaptive way using log-harmonic distance restraints and automated weighting [57].

## Supporting Information

S1 Fig Top-down analysis of the glycosylation sites present on the three main proteoforms of the FAM20*pglHpptA* strain. For each proteoform 3 panels describe: (A) A representative top-down ETD MS/MS Orbitrap spectrum that has been performed on a single charge state of PilE; (B) shows the full fragmentation maps resulting from PTM assignment for individual experiments perform on single charge states of each proteoform and (C) The combined full fragmentation map from all experiments in panel B. Details of the assigned ions can be found in S1 Table.
(TIF)

S2 Fig Transmission electron microscopy of negatively stained bacteria as previously described [36]. (A) LIM707; (B) LIM707*pglD*; (C) LIM354; (D) LIM534*pglD*; (E) FAM20; and (F) FAM20*pglD*.
(TIF)

S3 Fig Impact of pilin glycosylation on adhesion to human epithelial cells. Strains 8013, LIM707, LIM 534 and their corresponding *pglD* mutants were allowed to adhere to A549 human epithelial cells for 4 hours and the number of adherent bacteria analyzed. A non-piliated *pilD* mutant of the 8013 strain was used as a negative control. Average and standard deviations are indicated from three independent experiments.
(TIF)

S4 Fig Twitching motility in absence of glycosylation in the class II pilin expressing strains. (TIF)

S1 Table c/z fragment ions of the three proteoforms found in the FAM20*pglHpptA* strain. (XLSX)

## References

[1] Brandtzaeg P, van Deuren M. Classification and pathogenesis of meningococcal infections. Methods in molecular biology. 2012;799:21–35. 10.1007/978-1-61779-346-2_2 . 21993637

[2] Pelicic V. Type IV pili: e pluribus unum? Mol Microbiol. 2008;68(4):827–37. 10.1111/j.1365-2958.2008.06197.x 18399938

[3] Virji M, Heckels JE, Potts WJ, Hart CA, Saunders JR. Identification of epitopes recognized by monoclonal antibodies SM1 and SM2 which react with all pili of Neisseria gonorrhoeae but which differentiate between two structural classes of pili expressed by Neisseria meningitidis and the distribution of their encoding sequences in the genomes of Neisseria spp. Journal of general microbiology. 1989;135(12):3239–51. . 2483993 10.1099/00221287-135-12-3239

[4] Cehovin A, Winterbotham M, Lucidarme J, Borrow R, Tang CM, Exley RM, et al Sequence conservation of pilus subunits in Neisseria meningitidis. Vaccine. 2010;28(30):4817–26.: 10.1016/j.vaccine.2010.04.065 . 20457291

[5] Wormann ME, Horien CL, Bennett JS, Jolley KA, Maiden MC, Tang CM, et al Sequence, distribution and chromosomal context of class I and class II pilin genes of Neisseria meningitidis identified in whole genome sequences. BMC genomics. 2014;15:253 10.1186/1471-2164-15-253 24690385 PMC4023411

[6] Giltner CL, Nguyen Y, Burrows LL. Type IV pilin proteins: versatile molecular modules. Microbiology and molecular biology reviews: MMBR. 2012;76(4):740–72. 10.1128/MMBR.00035-12 23204365 PMC3510520

[7] Kellogg DS Jr., Cohen IR, Norins LC, Schroeter AL, Reising G. Neisseria gonorrhoeae. II. Colonial variation and pathogenicity during 35 months in vitro. J Bacteriol. 1968;96(3):596–605. 4979098 10.1128/jb.96.3.596-605.1968 PMC252347

[8] Melican K, Michea Veloso P, Martin T, Bruneval P, Dumenil G. Adhesion of Neisseria meningitidis to dermal vessels leads to local vascular damage and purpura in a humanized mouse model. PLoS Pathog. 2013;9(1):e1003139 10.1371/journal.ppat.1003139 23359320 PMC3554624

[9] Poolman JT, Hopman CT, Zanen HC. Immunogenicity of meningococcal antigens as detected in patient sera. Infect Immun. 1983;40(1):398–406. 6131872 10.1128/iai.40.1.398-406.1983 PMC264860

[10] Rotman E, Seifert HS. The Genetics of Neisseria Species. Annual review of genetics. 2014 10.1146/annurev-genet-120213-092007 . 25251852

[11] Davies JK, Harrison PF, Lin YH, Bartley S, Khoo CA, Seemann T, et al The use of high-throughput DNA sequencing in the investigation of antigenic variation: application to Neisseria species. PloS one. 2014;9(1):e86704 10.1371/journal.pone.0086704 24466206 PMC3899283

[12] Helm RA, Seifert HS. Frequency and rate of pilin antigenic variation of Neisseria meningitidis. J Bacteriol. 2010;192(14):3822–3. 10.1128/JB.00280-10 20472803 PMC2897326

[13] Caugant DA, Maiden MC. Meningococcal carriage and disease—population biology and evolution. Vaccine. 2009;27 Suppl 2:B64–70. 10.1016/j.vaccine.2009.04.061 19464092 PMC2719693

[14] Gault J, Malosse C, Dumenil G, Chamot-Rooke J. A combined mass spectrometry strategy for complete posttranslational modification mapping of Neisseria meningitidis major pilin. Journal of mass spectrometry: JMS. 2013;48(11):1199–206. 10.1002/jms.3262 . 24259208

[15] Hegge FT, Hitchen PG, Aas FE, Kristiansen H, Lovold C, Egge-Jacobsen W, et al Unique modifications with phosphocholine and phosphoethanolamine define alternate antigenic forms of Neisseria gonorrhoeae type IV pili. Proc Natl Acad Sci U S A. 2004;101(29):10798–803. . 15249686 10.1073/pnas.0402397101 PMC490014

[16] Stimson E, Virji M, Makepeace K, Dell A, Morris HR, Payne G, et al Meningococcal pilin: a glycoprotein substituted with digalactosyl 2,4-diacetamido-2,4,6-trideoxyhexose. Mol Microbiol. 1995;17(6):1201–14. . 8594338 10.1111/j.1365-2958.1995.mmi_17061201.x

[17] Takahashi H, Yanagisawa T, Kim KS, Yokoyama S, Ohnishi M. Meningococcal PilV potentiates Neisseria meningitidis type IV pilus-mediated internalization into human endothelial and epithelial cells. Infect Immun. 2012;80(12):4154–66. 10.1128/IAI.00423-12 22988016 PMC3497409

[18] Chamot-Rooke J, Rousseau B, Lanternier F, Mikaty G, Mairey E, Malosse C, et al Alternative Neisseria spp. type IV pilin glycosylation with a glyceramido acetamido trideoxyhexose residue. Proc Natl Acad Sci U S A. 2007;104(37):14783–8. . 17804791 10.1073/pnas.0705335104 PMC1976187

[19] Jennings MP, Virji M, Evans D, Foster V, Srikhanta YN, Steeghs L, et al Identification of a novel gene involved in pilin glycosylation in Neisseria meningitidis. Mol Microbiol. 1998;29(4):975–84. . 9767566 10.1046/j.1365-2958.1998.00962.x

[20] Borud B, Viburiene R, Hartley MD, Paulsen BS, Egge-Jacobsen W, Imperiali B, et al Genetic and molecular analyses reveal an evolutionary trajectory for glycan synthesis in a bacterial protein glycosylation system. Proc Natl Acad Sci U S A. 2011;108(23):9643–8. 10.1073/pnas.1103321108 21606362 PMC3111294

[21] Borud B, Anonsen JH, Viburiene R, Cohen EH, Samuelsen AB, Koomey M. Extended glycan diversity in a bacterial protein glycosylation system linked to allelic polymorphisms and minimal genetic alterations in a glycosyltransferase gene. Mol Microbiol. 2014 10.1111/mmi.12789 . 25213144

[22] Kahler CM, Martin LE, Tzeng YL, Miller YK, Sharkey K, Stephens DS, et al Polymorphisms in pilin glycosylation Locus of Neisseria meningitidis expressing class II pili. Infect Immun. 2001;69(6):3597–604. . 11349019 10.1128/IAI.69.6.3597-3604.2001 PMC98345

[23] Aas FE, Vik A, Vedde J, Koomey M, Egge-Jacobsen W. Neisseria gonorrhoeae O-linked pilin glycosylation: functional analyses define both the biosynthetic pathway and glycan structure. Mol Microbiol. 2007;65(3):607–24. 10.1111/j.1365-2958.2007.05806.x 17608667 PMC1976384

[24] Power PM, Seib KL, Jennings MP. Pilin glycosylation in Neisseria meningitidis occurs by a similar pathway to wzy-dependent O-antigen biosynthesis in Escherichia coli. Biochem Biophys Res Commun. 2006;347(4):904–8. . 16870136 10.1016/j.bbrc.2006.06.182

[25] Bentley SD, Vernikos GS, Snyder LA, Churcher C, Arrowsmith C, Chillingworth T, et al Meningococcal genetic variation mechanisms viewed through comparative analysis of serogroup C strain FAM18. PLoS genetics. 2007;3(2):e23 10.1371/journal.pgen.0030023 17305430 PMC1797815

[26] Dyer DW, McKenna W, Woods JP, Sparling PF. Isolation by streptonigrin enrichment and characterization of a transferrin-specific iron uptake mutant of Neisseria meningitidis. Microbial pathogenesis. 1987;3(5):351–63. . 3143887 10.1016/0882-4010(87)90005-2

[27] Gault J, Malosse C, Machata S, Millien C, Podglajen I, Ploy MC, et al Complete posttranslational modification mapping of pathogenic Neisseria meningitidis pilins requires top-down mass spectrometry. Proteomics. 2014;14(10):1141–51. 10.1002/pmic.201300394 24459079 PMC4201860

[28] Smith LM, Kelleher NL, Consortium for Top Down P. Proteoform: a single term describing protein complexity. Nat Methods. 2013;10(3):186–7. 10.1038/nmeth.2369 23443629 PMC4114032

[29] Naessan CL, Egge-Jacobsen W, Heiniger RW, Wolfgang MC, Aas FE, Rohr A, et al Genetic and functional analyses of PptA, a phospho-form transferase targeting type IV pili in Neisseria gonorrhoeae. J Bacteriol. 2008;190(1):387–400. . 17951381 10.1128/JB.00765-07 PMC2223744

[30] Warren MJ, Jennings MP. Identification and characterization of pptA: a gene involved in the phase-variable expression of phosphorylcholine on pili of Neisseria meningitidis. Infect Immun. 2003;71(12):6892–8. . 14638777 10.1128/IAI.71.12.6892-6898.2003 PMC308910

[31] Viburiene R, Vik A, Koomey M, Borud B. Allelic variation in a simple sequence repeat element of neisserial pglB2 and its consequences for protein expression and protein glycosylation. J Bacteriol. 2013;195(15):3476–85. 10.1128/JB.00276-13 23729645 PMC3719539

[32] Jen FE, Warren MJ, Schulz BL, Power PM, Swords WE, Weiser JN, et al Dual pili post-translational modifications synergize to mediate meningococcal adherence to platelet activating factor receptor on human airway cells. PLoS Pathog. 2013;9(5):e1003377 10.1371/journal.ppat.1003377 23696740 PMC3656113

[33] Jennings MP, Jen FE, Roddam LF, Apicella MA, Edwards JL. Neisseria gonorrhoeae pilin glycan contributes to CR3 activation during challenge of primary cervical epithelial cells. Cell Microbiol. 2011;13(6):885–96. 10.1111/j.1462-5822.2011.01586.x 21371235 PMC3889163

[34] Merz AJ, So M, Sheetz MP. Pilus retraction powers bacterial twitching motility. Nature. 2000;407(6800):98–102. 10.1038/35024105 . 10993081

[35] Parge HE, Forest KT, Hickey MJ, Christensen DA, Getzoff ED, Tainer JA. Structure of the fibre-forming protein pilin at 2.6 A resolution. Nature. 1995;378(6552):32–8. . 7477282 10.1038/378032a0

[36] Chamot-Rooke J, Mikaty G, Malosse C, Soyer M, Dumont A, Gault J, et al Posttranslational modification of pili upon cell contact triggers N. meningitidis dissemination. Science. 2011;331(6018):778–82. 10.1126/science.1200729 . 21311024

[37] Mortezaei N, Singh B, Bullitt E, Uhlin BE, Andersson M. P-fimbriae in the presence of anti-PapA antibodies: new insight of antibodies action against pathogens. Scientific reports. 2013;3:3393 10.1038/srep03393 24292100 PMC3848023

[38] Borud B, Aas FE, Vik A, Winther-Larsen HC, Egge-Jacobsen W, Koomey M. Genetic, structural, and antigenic analyses of glycan diversity in the O-linked protein glycosylation systems of human Neisseria species. J Bacteriol. 2010;192(11):2816–29. 10.1128/JB.00101-10 20363948 PMC2876500

[39] Shewell LK, Ku SC, Schulz BL, Jen FE, Mubaiwa TD, Ketterer MR, et al Recombinant truncated AniA of pathogenic Neisseria elicits a non-native immune response and functional blocking antibodies. Biochem Biophys Res Commun. 2013;431(2):215–20. 10.1016/j.bbrc.2012.12.132 23313483 PMC4326246

[40] Boslego JW, Tramont EC, Chung RC, McChesney DG, Ciak J, Sadoff JC, et al Efficacy trial of a parenteral gonococcal pilus vaccine in men. Vaccine. 1991;9(3):154–62. . 1675029 10.1016/0264-410x(91)90147-x

[41] Tramont EC, Sadoff JC, Boslego JW, Ciak J, McChesney D, Brinton CC, et al Gonococcal pilus vaccine. Studies of antigenicity and inhibition of attachment. The Journal of clinical investigation. 1981;68(4):881–8. 6116723 10.1172/JCI110343 PMC370875

[42] Power PM, Roddam LF, Rutter K, Fitzpatrick SZ, Srikhanta YN, Jennings MP. Genetic characterization of pilin glycosylation and phase variation in Neisseria meningitidis. Mol Microbiol. 2003;49(3):833–47. . 12864863 10.1046/j.1365-2958.2003.03602.x

[43] Mayer LW. Rates in vitro changes of gonococcal colony opacity phenotypes. Infect Immun. 1982;37(2):481–5. 6126433 10.1128/iai.37.2.481-485.1982 PMC347559

[44] Richardson AR, Stojiljkovic I. Mismatch repair and the regulation of phase variation in Neisseria meningitidis. Mol Microbiol. 2001;40(3):645–55. . 11359570 10.1046/j.1365-2958.2001.02408.x

[45] Lamelas A, Harris SR, Roltgen K, Dangy JP, Hauser J, Kingsley RA, et al Emergence of a New Epidemic Neisseria meningitidis Serogroup A Clone in the African Meningitis Belt: High-Resolution Picture of Genomic Changes That Mediate Immune Evasion. mBio. 2014;5(5). 10.1128/mBio.01974-14 . 25336458 PMC4212839

[46] Vik A, Aspholm M, Anonsen JH, Borud B, Roos N, Koomey M. Insights into type IV pilus biogenesis and dynamics from genetic analysis of a C-terminally tagged pilin: a role for O-linked glycosylation. Mol Microbiol. 2012;85(6):1166–78. 10.1111/j.1365-2958.2012.08166.x . 22882659

[47] Harvey H, Kus JV, Tessier L, Kelly J, Burrows LL. Pseudomonas aeruginosa D-arabinofuranose biosynthetic pathway and its role in type IV pilus assembly. J Biol Chem. 2011;286(32):28128–37. 10.1074/jbc.M111.255794 21676874 PMC3151058

[48] VanDyke DJ, Wu J, Logan SM, Kelly JF, Mizuno S, Aizawa S, et al Identification of genes involved in the assembly and attachment of a novel flagellin N-linked tetrasaccharide important for motility in the archaeon Methanococcus maripaludis. Mol Microbiol. 2009;72(3):633–44. 10.1111/j.1365-2958.2009.06671.x . 19400781

[49] Bernard SC, Simpson N, Join-Lambert O, Federici C, Laran-Chich MP, Maissa N, et al Pathogenic Neisseria meningitidis utilizes CD147 for vascular colonization. Nature medicine. 2014;20(7):725–31. 10.1038/nm.3563 . 24880614 PMC7095922

[50] Ke SH, Madison EL. Rapid and efficient site-directed mutagenesis by single-tube 'megaprimer' PCR method. Nucleic Acids Res. 1997;25(16):3371–2. 9241254 10.1093/nar/25.16.3371 PMC146891

[51] Marceau M, Beretti JL, Nassif X. High adhesiveness of encapsulated Neisseria meningitidis to epithelial cells is associated with the formation of bundles of pili. Mol Microbiol. 1995;17(5):855–63. . 8596435 10.1111/j.1365-2958.1995.mmi_17050855.x

[52] Eugene E, Hoffmann I, Pujol C, Couraud PO, Bourdoulous S, Nassif X. Microvilli-like structures are associated with the internalization of virulent capsulated Neisseria meningitidis into vascular endothelial cells. J Cell Sci. 2002;115(Pt 6):1231–41. . 11884522 10.1242/jcs.115.6.1231

[53] Sali A, Blundell TL. Comparative protein modelling by satisfaction of spatial restraints. Journal of Molecular Biology. 1993;234(3):779–815. 10.1006/jmbi.1993.1626 . 8254673

[54] Shen M-Y, Sali A. Statistical potential for assessment and prediction of protein structures. Protein science: a publication of the Protein Society. 2006;15(11):2507–24. 10.1110/ps.062416606 17075131 PMC2242414

[55] Brünger AT, Adams PD, Clore GM, DeLano WL, Gros P, Grosse-Kunstleve RW, et al Crystallography & NMR system: A new software suite for macromolecular structure determination. Acta crystallographica Section D, Biological crystallography. 1998;54(Pt 5):905–21. . 9757107 10.1107/s0907444998003254

[56] Linge JP, Williams MA, Spronk CA, Bonvin AM, Nilges M. Refinement of protein structures in explicit solvent. Proteins. 2003;50(3):496–506. . 12557191 10.1002/prot.10299

[57] Nilges M, Bernard A, Bardiaux B, Malliavin T, Habeck M, Rieping W. Accurate NMR structures through minimization of an extended hybrid energy. Structure. 2008;16(9):1305–12. 10.1016/j.str.2008.07.008 18786394

[58] Nassif X, Lowy J, Stenberg P, O'Gaora P, Ganji A, So M. Antigenic variation of pilin regulates adhesion of Neisseria meningitidis to human epithelial cells. Mol Microbiol. 1993;8(4):719–25. . 8332064 10.1111/j.1365-2958.1993.tb01615.x

[59] Geoffroy MC, Floquet S, Metais A, Nassif X, Pelicic V. Large-scale analysis of the meningococcus genome by gene disruption: resistance to complement-mediated lysis. Genome Res. 2003;13(3):391–8. . 12618369 10.1101/gr.664303 PMC430250

