# Supplementary figures and images for: Neisseria meningitidis Type IV Pili Composed of Sequence Invariable Pilins Are Masked by Multisite Glycosylation
Source: PLoS Pathog. 2015 Sep 14;11(9):e1005162. doi: 10.1371/journal.ppat.1005162 (PMC4569582; doi:10.1371/journal.ppat.1005162)

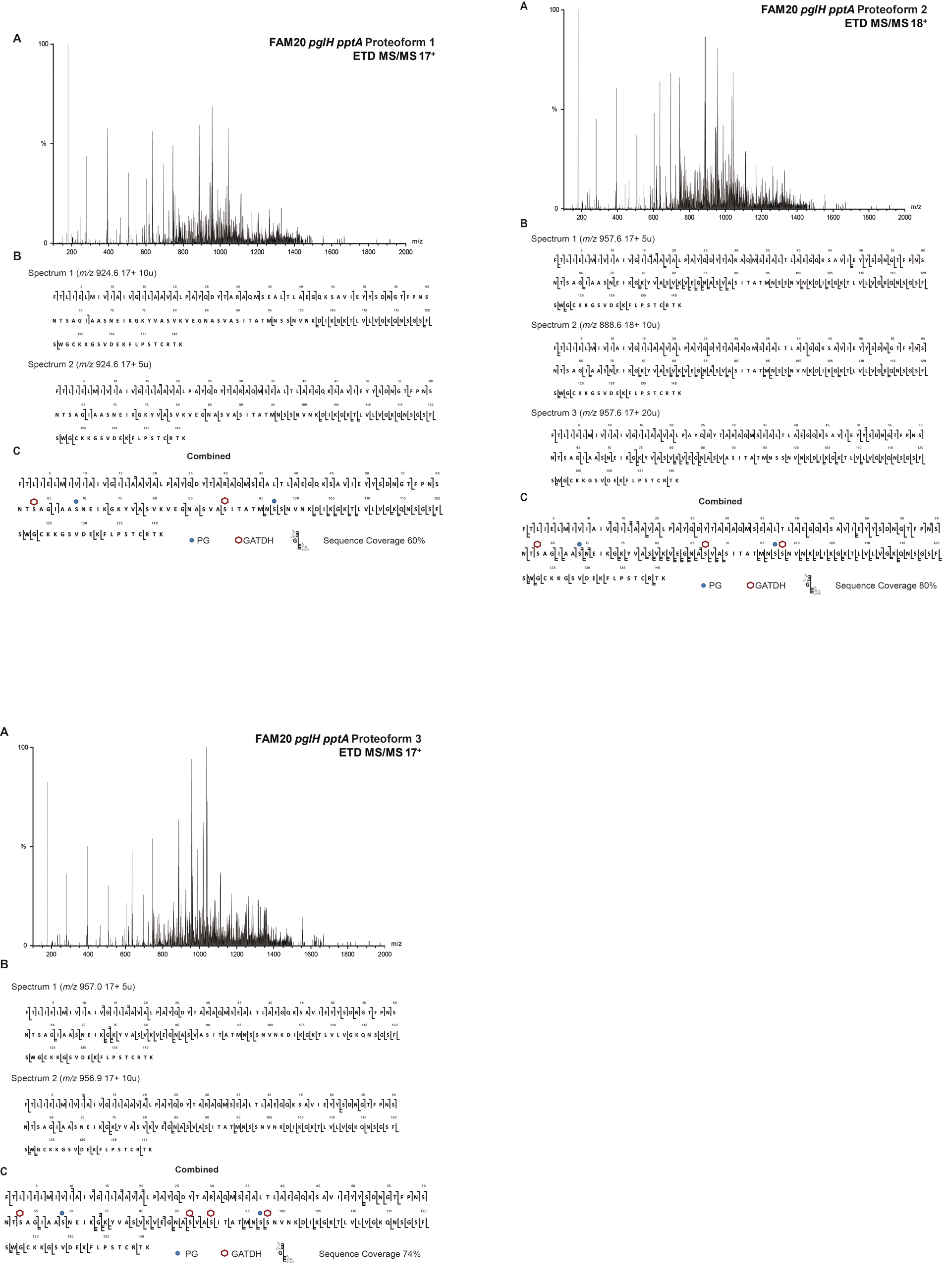

Supplement: S1 Fig — For each proteoform 3 panels describe: (A) A representative top-down ETD MS/MS Orbitrap spectrum that has been performed on a single charge state of PilE; (B) shows the full fragmentation maps resulting from PTM assignment for individual experiments perform on single charge states of each proteoform and (C) The combined full fragmentation map from all experiments in panel B. Details of the assigned ions can be found in S1 Table. (TIF) [file ppat.1005162.s001.tif]

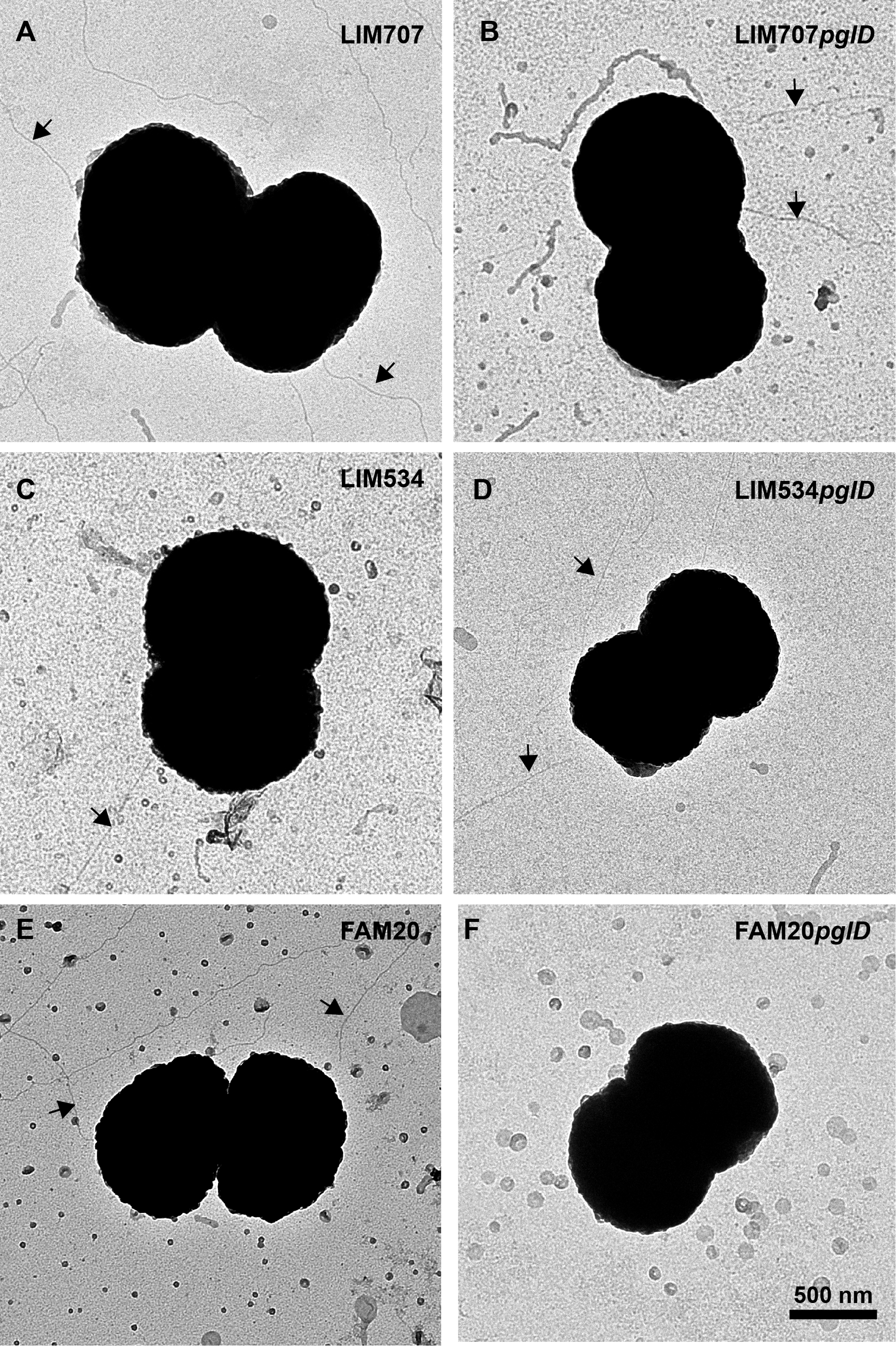

Supplement: S2 Fig — (A) LIM707; (B) LIM707pglD; (C) LIM354; (D) LIM534pglD; (E) FAM20; and (F) FAM20pglD. (TIF) [file ppat.1005162.s002.tif]

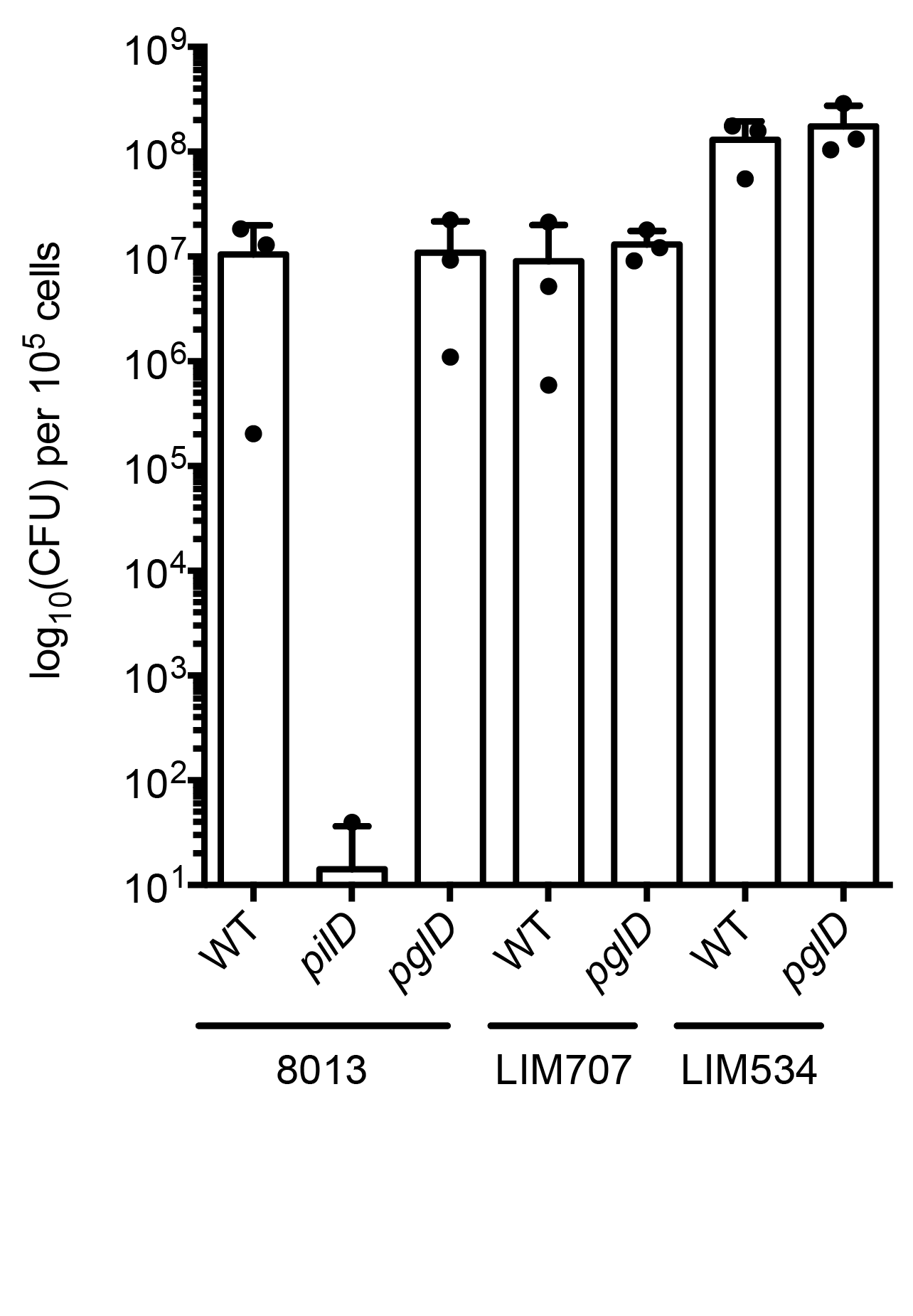

Supplement: S3 Fig — Strains 8013, LIM707, LIM 534 and their corresponding pglD mutants were allowed to adhere to A549 human epithelial cells for 4 hours and the number of adherent bacteria analyzed. A non-piliated pilD mutant of the 8013 strain was used as a negative control. Average and standard deviations are indicated from three independent experiments. (TIF) [file ppat.1005162.s003.tif]

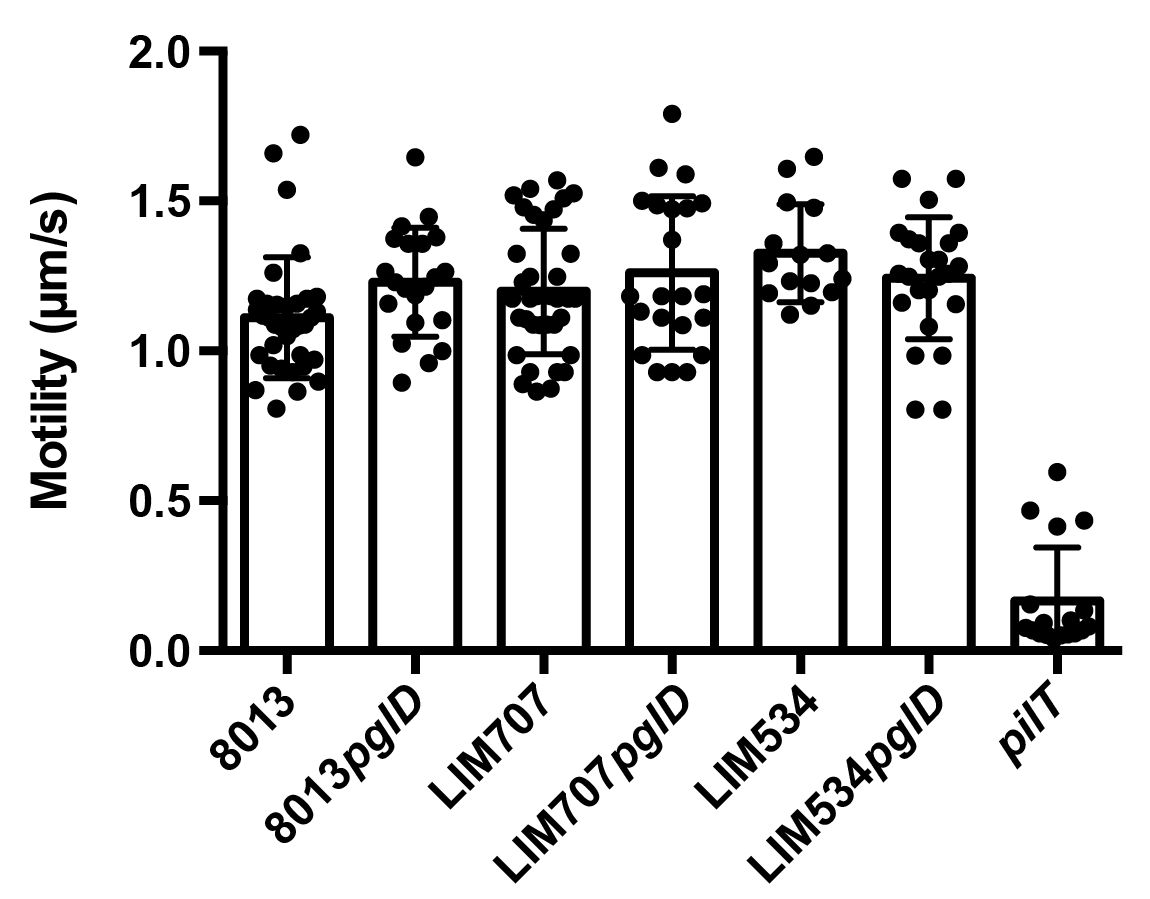

Supplement: S4 Fig — (TIF) [file ppat.1005162.s004.tif]
